# Tribomechanical Properties of PVA/Nomex^®^ Composite Hydrogels for Articular Cartilage Repair

**DOI:** 10.3390/gels10080514

**Published:** 2024-08-03

**Authors:** Francisco Santos, Carolina Marto-Costa, Ana Catarina Branco, Andreia Sofia Oliveira, Rui Galhano dos Santos, Madalena Salema-Oom, Roberto Leonardo Diaz, Sophie Williams, Rogério Colaço, Célio Figueiredo-Pina, Ana Paula Serro

**Affiliations:** 1 Centro de Química Estrutural (CQE), Institute of Molecular Sciences, Department of Chemical Engineering, Instituto Superior Técnico, University of Lisbon, Av. Rovisco Pais 1, 1049-001 Lisbon, Portugal; f.dos.santos@tecnico.ulisboa.pt (F.S.); carolina.marto.costa@tecnico.ulisboa.pt (C.M.-C.); ana.branco@tecnico.ulisboa.pt (A.C.B.); andreia.oliveira@tecnico.ulisboa.pt (A.S.O.); 2 Egas Moniz Center for Interdisciplinary Research (CiiEM), Egas Moniz School of Health & Science, Monte da Caparica, 2829-511 Almada, Portugal; moom@egasmoniz.edu.pt; 3 Escola Superior de Tecnologia de Setúbal, Instituto Politécnico de Setúbal, 2910-761 Setúbal, Portugal; 4 Instituto de Engenharia Mecânica (IDMEC), Department of Mechanical Engineering, Instituto Superior Técnico, University of Lisbon, Av. Rovisco Pais 1, 1049-001 Lisbon, Portugal; rogerio.colaco@tecnico.ulisboa.pt; 5 CERENA—Centre for Natural Resources and the Environment, Instituto Superior Técnico, University of Lisbon, Av. Rovisco Pais, 1049-001 Lisboa, Portugal; rui.galhano@tecnico.ulisboa.pt; 6 Institute of Medical and Biological Engineering, School of Mechanical Engineering, University of Leeds, Woodhouse, Leeds LS2 9JT, UK; r.leonardodiaz@leeds.ac.uk (R.L.D.); s.d.williams@leeds.ac.uk (S.W.); 7 CeFEMA—Center of Physiscs and Engineering of Advanced Materials, Instituto Superior Técnico, University of Lisbon, Av. Rovisco Pais 1, 1049-001 Lisbon, Portugal

**Keywords:** poly(vinyl alcohol), aramid fibers reinforcement, hydrogels, polymeric composites, tribomechanical properties, hip simulation tests

## Abstract

Due to the increasing prevalence of articular cartilage diseases and limitations faced by current therapeutic methodologies, there is an unmet need for new materials to replace damaged cartilage. In this work, poly(vinyl alcohol) (PVA) hydrogels were reinforced with different amounts of Nomex^®^ (known for its high mechanical toughness, flexibility, and resilience) and sterilized by gamma irradiation. Samples were studied concerning morphology, chemical structure, thermal behavior, water content, wettability, mechanical properties, and rheological and tribological behavior. Overall, it was found that the incorporation of aramid nanostructures improved the hydrogel’s mechanical performance, likely due to the reinforcement’s intrinsic strength and hydrogen bonding to PVA chains. Additionally, the sterilization of the materials also led to superior mechanical properties, possibly related to the increased crosslinking density through the hydrogen bonding caused by the irradiation. The water content, wettability, and tribological performance of PVA hydrogels were not compromised by either the reinforcement or the sterilization process. The best-performing composite, containing 1.5% wt. of Nomex^®^, did not induce cytotoxicity in human chondrocytes. Plugs of this hydrogel were inserted in porcine femoral heads and tested in an anatomical hip simulator. No significant changes were observed in the hydrogel or cartilage, demonstrating the material’s potential to be used in cartilage replacement.

## 1. Introduction

Articular cartilage is a connective tissue present in the joints, characterized by the lack of nerves and blood vessels. Its structure primarily comprises a dense extracellular matrix consisting mainly of collagen fibers and proteoglycans and houses the single cell type present, the chondrocytes [1]. This tissue is responsible for covering the surface of joints, bearing and distributing the loads that result from daily activities [2,3]. Additionally, it plays a crucial role in keeping low friction and minimum wear within the articulating surfaces [4].

The intrinsic healing capacity of articular cartilage is severely limited due to the restricted proliferative ability of chondrocytes. As such, it can suffer deterioration and wear caused by injury or pathologies, resulting in painful symptoms and, ultimately, in loss of joint function [1]. Osteoarthritis, the most common joint pathology, impacts over 240 million people worldwide and is recognized as the primary cause of activity limitation in adults [5]. Furthermore, damaged cartilage is notably pronounced in overweight and elderly populations, both of which are burgeoning trends in global demographics [6].

Various strategies have been used to alleviate symptoms and restore joint functionality. These range from the use of physiotherapy and anti-inflammatory medications in the early stages of the pathologies to several surgical options used in more severe stages. The latter include cartilage restoration techniques as chondrocyte implantation, microfracture, and autografts/allografts [7]. Regrettably, these approaches have shown high failure rates, with up to 40% of treatments not being effective for more than 15 years [8,9]. Several attempts have been made to produce synthetic materials intended to mimic and/or repair the native cartilage tissues. Currently, it is already possible to find in the market products like MaioRegen [10], a biomimetic scaffold composed of collagen and hydroxyapatite that aims to restore osteochondral joint defects. Cartiva [11], an osteochondral defect repair implant based on poly(vinyl alcohol) (PVA) specifically designed to treat the hallux rigidus, or BioPoly [12], an implant material with self-lubricating properties resultant from the combination of hyaluronic acid with ultra-high-molecular-weight polyethylene, which can be used for substitution of the great toe or knee cartilage. Despite the good clinical outcome in patients where these solutions were applied, challenges still remain regarding their mechanical integrity under load-bearing conditions, with further studies being needed [13,14].

There are many studies in the literature reporting the development of new materials for this purpose. However, most of them present inadequate mechanical and/or tribological properties [7,15].

Hydrogels have recently been gaining attention in this field, owing to their similarity to natural soft tissues [16,17]. They present a three-dimensional crosslinked polymer network able to retain a high water content, with tuneable porosity and lubrication capacity [18]. Among these biomaterials, PVA hydrogels have emerged as a particularly interesting alternative for articular cartilage substitution [19,20]. They are biocompatible, demonstrate favorable swelling properties, and exhibit low friction coefficients [21,22]. However, it has been reported that PVA hydrogels usually lack elastic moduli and toughness capable of adequately mimicking the native articular cartilage. Therefore, these materials often do not survive extended exposure to the expected forces in load-bearing joints [23]. Nevertheless, the characteristics of these hydrogels can be improved by altering the production method and crosslinking levels, as well as integrating other materials, such as different polymers and fillers [24,25]. The reinforcement of materials for cartilage substitution/repair have been attempted by a wide number of authors regarding not only PVA-based hydrogels but also others. The incorporation of fillers like graphene oxide [26], hydroxyapatite [27], nanocellulose [28], and aramid [29] have been explored in the last years.

Aramid fibers, characterized by their exceptional mechanical properties and chemical and thermal resistance are high-performance aromatic polyamides extensively used in advanced composites as reinforcement materials [30,31,32]. The chemical exfoliation of these fibers has been demonstrated to produce aramid nanofibers (ANFs) that inherit the macrofibers’ superb mechanical and thermal properties [32]. This is attractive because, although widely used to reinforce polymers, aramid fibers show poor adhesion to the polymeric matrix, leading to phase separation and a limited increase in the mechanical properties of composites [33]. In comparison, their nanoscale leads to a high surface area, which results in stronger interactions with the matrix through hydrogen bonding or pi stacking, giving rise to an exceptional strengthening of the polymer matrix [33].

Recently, ANFs have been shown to be good candidates for the reinforcement of PVA-based materials, including hydrogels, owing to significant hydrogen bonding between the two polymers [33]. Guan et al. [34] fabricated PVA/para-ANF nanocomposite films achieving tensile strength and toughness values which were 79.2% and 148.8% higher than those of PVA alone, respectively. Also, Guo et al. [35] successfully reinforced PVA-based hydrogels with para-ANFs, which showed enhanced mechanical and antibacterial properties for application in wound dressings. Furthermore, Xu et al. [29] developed synthetic cartilage using these hydrogel composites and observed a mechanical performance equal to that of cartilage tissues. The material presented adequate water content and cytocompatibility.

Even though ANFs can be produced from both meta-aramid and para-aramid fibers [36], due to the superior mechanical properties of para-aramids, most research focuses solely on these nanofibers for the reinforcement of PVA-based materials. Nonetheless, meta-aramid fibers, like poly (m-phenylene isophthalamide) (PMIA), commercially known as Nomex^®^, demonstrate high mechanical toughness, flexibility, and resilience [37]. Additionally, these possess a higher elasticity and may be cheaper when compared to para-aramid fibers [30,32]. 

To clinically translate hydrogels for implantation in the body, effective sterilization methodologies which do not affect the material’s biocompatibility need to be established to guarantee their biological safety [38,39]. Ideally, sterilization is the ultimate phase in the production of any biomaterial, and thus its effect on the material’s surface and properties should be clearly understood and assessed [40]. This is of particular importance in the case of hydrogels due to their sensitivity towards various sterilization agents, such as heat [41] and radiation [24].

The aim of this work is to investigate the potential of PVA-based composite hydrogels reinforced with different contents of PMIA and sterilized with gamma radiation to be used in hyaline cartilage repair, the most common in load-bearing joints. The study hypothesis is that both the incorporation of the nanofibers and the irradiation procedure enhance the tribomechanical properties. The materials were characterized regarding micromorphology, chemical structure, thermal behavior, water content, wettability, mechanical properties, and rheological, and tribological behavior. The biocompatibility of the best material was assessed through cytotoxicity assays using human chondrocytes. Finally, this work was pioneering in its use of an anatomical hip simulator to test the possibility of using hydrogel as a graft to repair defects in hip joint cartilage.

## 2. Results and Discussion

### 2.1. Morphology

The dissociation of PMIA fibers into particles was evaluated using atomic force microscopy (AFM) measurements. AFM images of a diluted PMIA dispersion indicate the presence of spherical nanostructures of about 40 to 300 nm (Figure S1). In contrast to previous works ([29,33,36], [41]), the exfoliation of PMIA fibers in DMSO in the presence of KOH did not result in a nanofiber suspension. In this case, the resultant particles observed may be attributed to the increased concentration of aramid fibers and decreased mass ratio of KOH to fibers used compared to what has been reported for para-aramids [33,36,41]. Although these conditions may not be adequate to delaminate the macro-dimensioned PMIA fibers into nanofibers with dimensions described for para-aramids (5–30 nm in diameter) and also led to the formation of particles, the process implemented here allows us to obtain dispersions of aramid particles with significantly higher concentrations (up to two times higher) [29,33,41].

**Figure 1:**
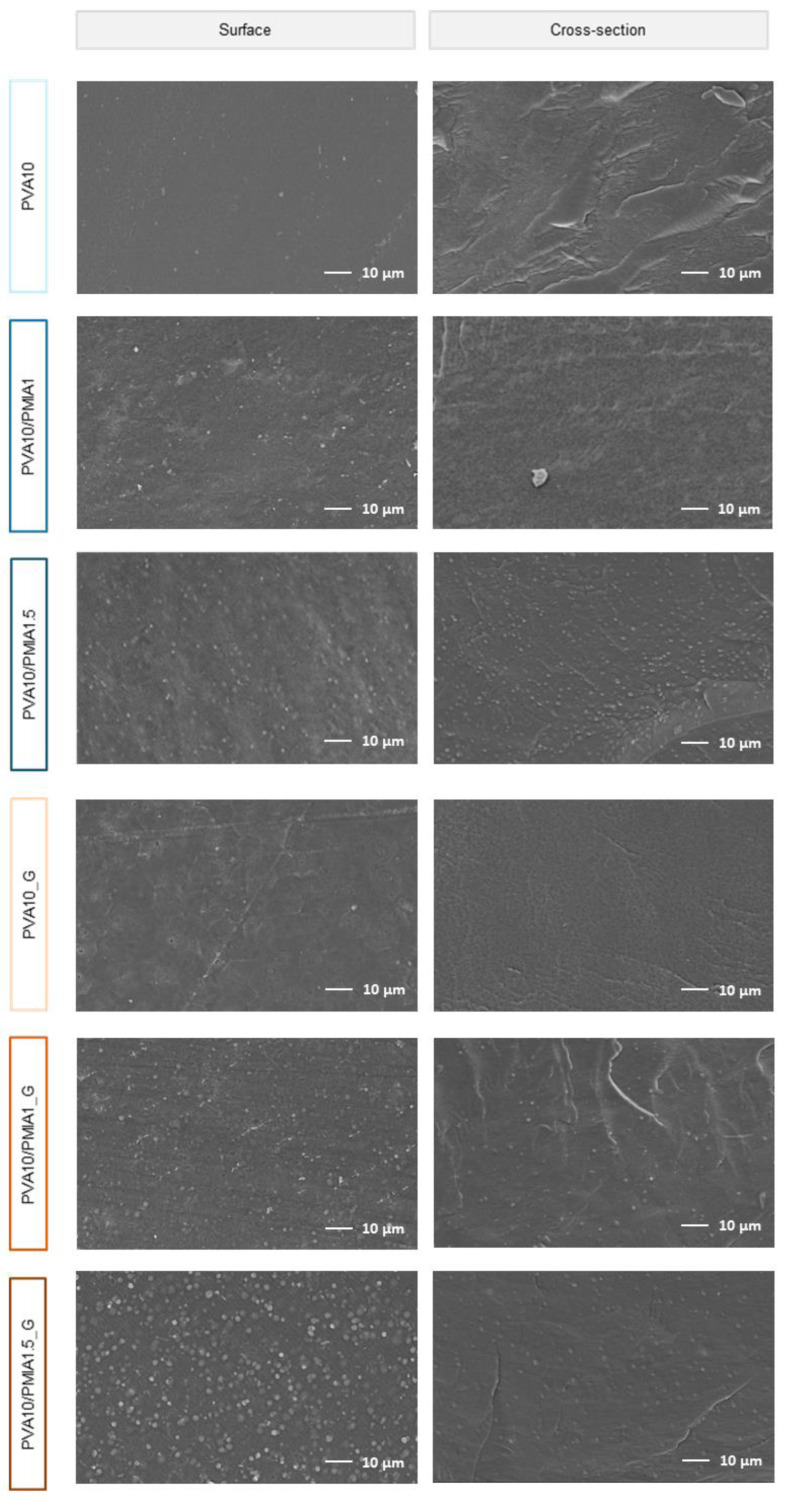
SEM images of the surface and cross-section of hydrogel samples, acquired with 1000× magnification.

Scanning electron microscopy (SEM) images of the surface and cross-section of the produced materials, both before and after sterilization, are depicted in Figure 1. All hydrogels present a compact structure free of pores. All composite hydrogels show round structures with a size ≤1.5 µm, especially visible in the materials with 1.5% reinforcement. Energy dispersive spectroscopy (EDS) analysis (Figure S2) demonstrated that these structures contain a higher amount of nitrogen than the surrounding material. Considering that the sole source of nitrogen in these materials is PMIA, it is likely that these observed structures correspond to the PMIA particles that remain separate and distinct. 

The non-porous nature of the hydrogels may be considered a disadvantage when developing new hydrogels for cartilage repair, given that it has been found that a more porous and permeable structure enhances the migration of cells and provides better mechanical anchorage onto the material, promoting its in vivo integration [42]. Additionally, microporosity can simulate the natural fluid exudation and pressurization processes observed in cartilage while also enhancing its capacity for load dissipation [43]. Even so, various reports have shown that hydrogels can mimic the necessary characteristics of natural articular cartilage even with a more compact structure, suggesting that the material can perform the desired function without porosity [24,29,44,45].

### 2.2. Chemical and Thermal Characterization

Fourier-transform infrared (FTIR) and thermogravimetric analysis (TGA) were used to evaluate the interactions among the PVA polymer chains and the reinforcing PMIA, as well as the possible chemical changes that the gamma irradiation of the hydrogels may have caused. For the composite materials, only those with the higher content of PMIA (1.5%) were analyzed to facilitate the detection of eventual changes due to the presence of the reinforcement. 

The FTIR spectra of PVA10 and PVA/PMIA1.5, before and after exposure to gamma irradiation, are shown in Figure S3. In both the commercial PVA powder and the hydrogel PVA10, the typical vibrational peaks [15,46] occurred at 3272 cm^−1^ (intramolecular bonded O–H stretching), 2940 cm^−1^ and 2908 cm^−1^ (asymmetric and symmetric CH_2_ stretching modes, respectively), 1651 cm^−1^ (C=O stretching of residual vinyl acetate groups, as a consequence of the polymer synthesis), 1417 cm^−1^ and 1326 cm^−1^ (CH_2_ bending), 1142 cm^−1^ (C–O crystallinity), and 1089 cm^−1^ (C–O bond stretching, alcohol). The spectrum of the commercial PMIA fibers (Nomex^®^) showed expected absorption peaks assigned to the following vibrations [35]: 3307 cm^−1^ (N–H stretching, secondary amide); 1646 cm^−1^ (C=O stretching); 1603 cm^−1^ (C=C stretching of aromatic rings); 1528 cm^−1^ (N–H deformation and C–N stretching coupled modes); and 1241 cm^−1^ (N–C of aromatic rings stretching). Regarding the reinforced hydrogel PVA10/PMIA1.5, it is possible to observe that the spectrum presents peaks characteristic of its two components. Aside from the additive effect due to the presence of the two polymers, there was no notable change to the peaks observed that could provide evidence of the type of interactions between them. The spectra of the gamma-irradiated samples (PVA10_G and PVA10/PMIA1.5_G) showed no significant changes compared to the non-irradiated materials.

The TGA curves of non-irradiated and irradiated PVA10 and PVA10/PMIA1.5 are presented in Figure S4. Overall, the thermogravimetric curves are divided mainly into three stages, similar to previous studies of PVA and PVA-based samples [15,47]. During the first stage, up to 240 °C, the weight loss is associated with free and bound volatilization of water or residual solvents [48,49]. The second stage can be attributed to the PVA O-H group degradation (240–380 °C), while the last stage suggests an almost complete thermo-decomposition of PVA (380–480 °C), respectively [15,50]. 

A comparison of the PVA and composite hydrogels, PVA10 and PVA10/PMIA1.5, shows that PVA10 exhibits a T0.1 (the temperature at which the sample reports a 10% weight loss) of 334 ± 2 °C, while the composite material presents 319 ± 2 °C (insert in Figure S5). Such a decrease in the decomposition temperature (15 °C) is statistically significant (*p* < 0.01) and may be attributed to a reorganization of PVA chains in the presence of the PMIA. In fact, the PMIA fibers may interfere with the hydrogen bonding within the PVA chains, leading to a hydrogel structure that begins to degrade at a lower temperature compared to the pristine PVA. Although there was no notable change detected by FTIR that could provide evidence of the type of interactions between them, the literature suggests that the interaction of PVA and para-aramid nanofibers takes place via intermolecular hydrogen bonding, formed between the O–H groups of the PVA chains and both the N–H and C=O groups of the nanofibers [29,34]. As such, the presence of PMIA is likely to decrease the hydrogen bonds between PVA chains, impairing the formation of PVA domains with a more ordered structure. This is reflected by the decrease in the onset temperature of the initial weight loss of the reinforced hydrogel. However, at 600 °C, the weight loss for PVA10/PMIA1.5 was significantly lower than for PVA10, which may be attributed to the presence of the thermally resistive PMIA (which starts degrading at 430 °C [51]) and to its interaction with PVA [52].

Regarding the effect of gamma irradiation, a decrease in degradation temperature (T0.1) was observed from 334 ± 2 °C for PVA10 to 323 ± 6 °C for PVA10_G samples. However, this change was not statistically significant (*p* > 0.05). It is known that gamma irradiation can affect polymers in different extensions, depending on their molecular structure, composition, and radiation dose. The irradiation of an oxygen-free aqueous solution is known to induce the radiolysis of water, generating short-lived reactive intermediates, such as H• and •OH radicals [53]. In the presence of PVA, both species have been shown to abstract the hydrogen atom in the α-position to the hydroxyl group (–CH(OH)–), as well as from the methylene group (–CH_2_–), forming tertiary and secondary radicals, respectively. These PVA radicals may interact with one another by inter- and intramolecular crosslinking [52,53,54]. As no significant differences were found between PVA10 and PVA10_G in FTIR and TGA analysis, it is possible to infer that, in the present case, scission and/or crosslinking processes might have been involved in the breaking and forming of hydrogen bonds instead of chemical bonds since the latter would have a more pronounced effect on the TGA.

In the case of the composite materials, the irradiation led to a decrease of 62 °C in T0.1 (319 ± 2 °C for PVA10/PMIA1.5 and 257 ± 5 °C for PVA10/PMIA1.5_G, *p* < 0.001). As referred to above, during irradiation, the generation of radicals and the chain scission through *β*-fragmentation of the PVA polymer chains has been reported to occur [55]. However, it may not be accompanied by increased crosslinking if the PMIA structures hinder the interaction between PVA radicals. These findings are in agreement with those obtained by Martínez-Barrera et al. [56], who related lower values of crosslinking with decreased thermal stability. Despite the much lower value of T0.1 found for PVA10/PMIA1.5_G when compared to PVA10_G, a lower weight loss was observed at 600 °C, which may be attributed to the higher thermal resistance of PMIA compared to PVA. Although the effect of gamma irradiation on PMIA nanostructures has not been studied here, PMIA is widely known for its outstanding resistance to the deteriorating effects of gamma radiation, owing to the benzene rings within its structure [57]. Therefore, the degradation of the reinforced PVA upon irradiation was enhanced by the presence of PMIA.

### 2.3. Equilibrium Water Content and Wettability

**Figure 2:**
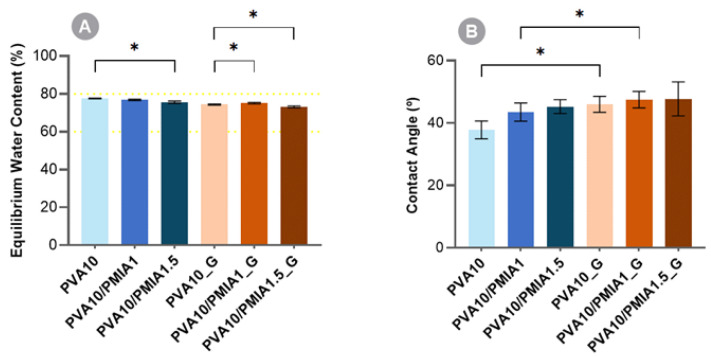
Equilibrium water content (**A**) and water contact angles (**B**) for PVA and PMIA-reinforced hydrogels. Data followed a normal distribution: Welch’s ANOVA and Dunnett’s C test or Student’s *t* test, *p* ≤ 0.05 (*).

Figure 2A shows the results of the equilibrium water content of the hydrogels. It can be observed that, for the non-irradiated hydrogels, the incorporation of the nanofibers led to a slight decrease in the water content of the materials. As suggested by Zhou et al. [58], this is likely due to the formation of hydrogen bonds between nanofibers and PVA polymer chains. These interactions would restrict the polymer chains’ mobility in the hydrogels’ network and, thus, reduce the amount of water that may enter the polymeric structure. Another factor that may contribute to this finding is the more hydrophobic nature of PMIA compared to PVA [59]. It has been reported that the hydrogels’ water content is largely dependent on the hydrophilicity of their functional groups, alongside the crosslinking density of the network [15,60]. 

Regarding the sterilized materials, it is possible to observe that the exposure of the hydrogels to gamma irradiation also led to a decrease in the water content, although this difference is not statistically significant. Still, this is in line with previous observations, where a reduction in the water content of PVA-based hydrogels was found to occur when the radiation dose was increased. This phenomenon was attributed to the enhancement of network crosslinking by irradiation [24,61].

The water content of natural articular cartilage depends on the type of cartilage and location [24], being within the 60–80% range. Taking into consideration that the materials produced are intended to be used as articular cartilage substitutes, ideally, this property should fall within the same range of values [62,63]. As such, all the hydrogels demonstrated adequate water content.

The samples’ wettability was determined through the captive bubble method. Figure 2B shows the water contact angles determined for the different hydrogels. All the produced materials are hydrophilic, presenting water contact angles ≤48°. The pristine PVA sample, PVA10, showed a contact angle of 38°, similar to what was previously reported for PVA-based hydrogels [24]. The incorporation of nanofibers led to a decrease in surface hydrophilicity, which may be a consequence of an eventual increase in surface roughness and/or of the less hydrophilic nature of the aramid compared to PVA, as referred to above. Aramid fibers have a reported contact angle of around 60° [64].

Sterilization with gamma radiation resulted in an increase in the hydrogels’ hydrophobicity. In particular, the non-reinforced hydrogel suffered a significant water contact angle increase (≈8°). The decrease in wettability is likely related to structural changes of the polymeric matrix induced by the radiation. According to Pohan et al. [65], irradiation may lead to the scission of PVA chains, exposing more polymer alkyl end groups, which are more hydrophobic compared to the hydroxyl groups. These authors also observed a decrease in hydroxyl groups percentage on the surface of the PVA irradiated hydrogels.

Values reported in the literature for the water contact angle with porcine knee cartilage measured under the same conditions are of the same order of magnitude as those obtained for the hydrogels studied in this work (38° ± 3°) [19].

### 2.4. Mechanical Properties

Compressive and tensile tests were carried out to evaluate the reinforcing effect of the PMIA and the effect of sterilization of the hydrogels on their mechanical performance.

**Figure 3:**
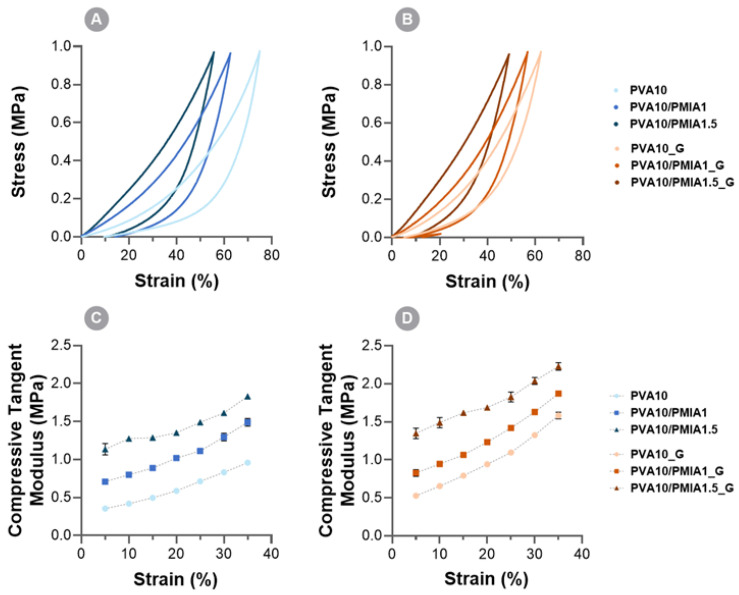
Typical compressive stress–strain curves for PVA- and PMIA-reinforced hydrogels before (**A**) and after (**C**) sterilization and corresponding values of compressive tangent modulus between 5% and 35% (**B** and **D**, respectively).

The typical compression stress–strain curves are depicted in Figure 3A,B. As can be seen, the incorporation of the treated PMIA led to a decrease in the compressibility of the samples, which is proportional to the amount of the reinforcement. The increase in stiffness relative to pristine PVA hydrogels is likely associated with the high intrinsic strength of PMIA [37]. Additionally, the good dispersion of the PMIA within the PVA network and the formation of intermolecular hydrogen bonding between PMIA and the PVA chains are thought to play a role in these properties [15,29,34].

The irradiation of the hydrogels further improved the compressive properties of the reinforced hydrogels. The irradiation of the non-reinforced PVA hydrogel also improved its compressive properties. The possible increase in the crosslinking degree of the PVA network, resulting from the scission and formation of new hydrogen bonds with the reorganization of the polymeric chains, likely led to the enhanced mechanical performance of the sterilized hydrogel [66]. In the literature, this improvement has been reported for hydrogels treated with up to doses of 100 kGy [24,57,66,67].

Regarding the composite hydrogels, the compressive behavior further improved upon irradiation. In fact, both materials became stiffer than the non-irradiated ones, leading to the conclusion that the reinforcing effect of the PMIA and the sterilization are synergistic. It should be highlighted that, although thermal stability (see Section 2.2) and mechanical behavior both depend on the polymer molecular structure, they may not be directly correlated. The obtained results show that both the addition of the reinforcement and the gamma irradiation alone tend to decrease the decomposition temperature but improve mechanical performance. Several other authors that studied the behavior of reinforced materials reached similar conclusions. This likely results from the fact that the factors that determine the thermal and the mechanical behavior depend on the temperature: for example, hydrogen bonds, which in the present work might play a key role in the improvement of the mechanical properties of the modified hydrogels, justifying the lower thermal resistance. It should be stressed that, although the FTIR spectra of the composite material does not evidence the establishment of covalent bonds between PMIA and PVA by irradiation, it is plausible that crosslinking occurs through the establishment of hydrogen bonds between PVA chains and/or PMIA and PVA. In fact, PVA has in its structure a wide number of hydroxyl (-OH) groups that can form hydrogen bonding, and PMIA presents amide (N-H) and carbonyl (C=O) groups that can act both as hydrogen donors (through the hydrogen in the former groups) and acceptors (through the oxygen in the latter).

The results displayed in Figure 3C,D denote that in all cases there is an increase in the compressive tangent moduli over the considered strain range. As reported for other hydrogels, it is expected that initially, the applied load is mainly supported by the liquid present within the polymer matrix. As the load is raised, a gradual exudation of water is expected to occur, leading the load to be transferred to the solid phase [46].

**Figure 4:**
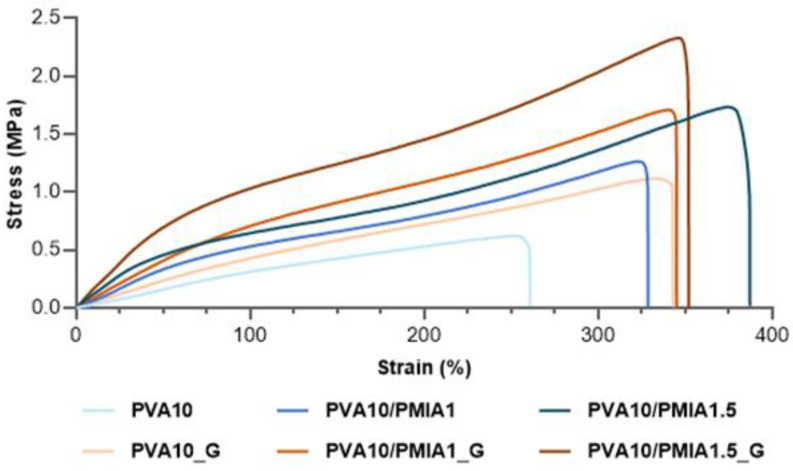
Typical tensile stress–strain curves of the PVA- and PMIA-reinforced hydrogels before and after sterilization.

**Figure 5:**
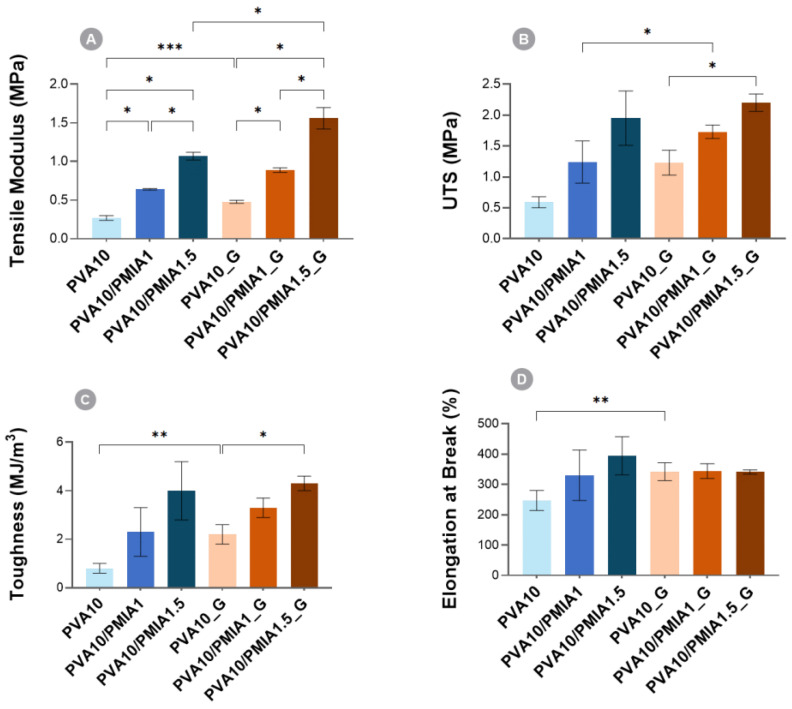
Tensile properties of PVA- and nanofiber-reinforced hydrogels: (**A**) tensile modulus; (**B**) UTS; (**C**) toughness; (**D**) elongation at break. When comparing non-irradiated vs. irradiated samples, data followed a normal distribution, except for tensile moduli and UTS: Student’s *t* test or Mann–Whitney U test. When comparing samples with different amounts of PMIA, data followed a normal distribution, except for tensile moduli: Welch’s ANOVA and Dunnett’s C test or Kruskal–Wallis tests adjusted with Bonferroni correction. *p* ≤ 0.05 (*), *p* ≤ 0.01 (**), *p* ≤ 0.001 (***).

Concerning the tensile properties, the typical stress–strain curves of the hydrogels are presented in Figure 4. The values of the tensile properties determined from the curves are displayed in Figure 5. A behavioral pattern similar to that found in compressive tests was observed. The increase in the content of treated PMIA led to improvements in stiffness (Figure 5A), strength (Figure 5B), and toughness (Figure 5C). These improvements are likely associated with the intrinsic properties of PMIA and the interaction between the two polymers [15,29,34,60]. Furthermore, the increase in the elongation at break of the reinforced samples indicates that, despite the incorporation of stiff polymer particles, the extensibility was not negatively impacted (Figure 5D) [15].

The sterilization of the samples also led to enhanced tensile properties. In particular, PVA10_G showed significantly higher tensile modulus and elongation at break compared to PVA10. As with the compressive properties, the additional crosslinking in the materials, reported to occur as a consequence of the ionizing radiation, is likely responsible for the improvements observed [24,67]. The combined effect of the PMIA reinforcement and the gamma irradiation led to an increase of 5.8× in the tensile modulus, 1.4× in elongation at break, 3.7× in ultimate tensile strength (UTS), and 5.4× in the toughness of PVA10/PMIA1.5_G when compared to PVA10. The tensile performance obtained here is significantly greater than that of PVA-based hydrogels containing other types of reinforcements, such as bacterial cellulose and graphene oxide [68,69,70,71]. However, the reinforcement of PVA-based hydrogels with para-aramid nanofibers showed superior results [29].

Considering the desired application, the mechanical behavior of the hydrogels ideally needs to be comparable to that of articular cartilage. However, as described by Oliveira et al. [24], the literature data concerning natural cartilage mechanical properties present a wide distribution due to differences in testing conditions, location, and type of the tissue. The compressive modulus reported for cartilage ranges between 0.1 and 10 MPa [72,73,74]. Although this is not directly comparable to the compressive tangent modulus, all the samples can be considered to show adequate values. Concerning the tensile behavior, values ranging from 0.5 to 30 MPa [72,75] and from 0.8 to 25 MPa [73,75,76] have been reported for cartilage’s tensile modulus and ultimate tensile strength, respectively. As can be seen, the tensile properties of the reinforced samples are also within the required range for this application. However, the enhanced mechanical performance of PVA10/PMIA1.5_G makes it the best potential candidate here. It should be stressed that although tensile properties are important for the material’s mechanical performance, they are not as critical as compressive ones since articular cartilage primarily experiences compressive loads during daily activities such as walking, running, and standing.

### 2.5. Rheological Behavior

**Figure 6:**
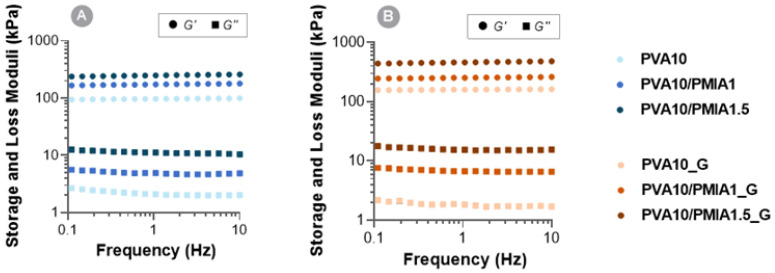
Storage (G′) and loss (G″) moduli of the PVA- and PMIA-reinforced hydrogels before (**A**) and after (**B**) sterilization as a function of the frequency (0.1–10 Hz).

The hydrogels’ viscoelastic response was investigated through their rheological characterization. Figure 6 shows the evolution of the storage (G′) and loss (G″) moduli of the samples over a frequency range of 0.1–10 Hz. It can be observed that all the hydrogels produced exhibited viscoelastic behavior. Nonetheless, the samples’ storage modulus is much greater than the loss modulus in all the tested frequency ranges, which shows the predominant elastic nature of the hydrogels and indicates complete network formation [77,78]. Moreover, both moduli do not show any significant dependence on the frequency within the range tested, revealing a stable gel-like behavior of the materials [19]. Overall, the storage and loss moduli values of these samples are within the same order of magnitude reported for PVA hydrogels [19,79].

The incorporation of treated PMIA led to an increase in both the storage and the loss moduli of the hydrogels. Considering that the storage modulus is directly related to the material stiffness [80], the increase in this value with an increasing amount of reinforcement is in accordance with what was observed in the compressive and tensile mechanical tests. The intrinsic strength of PMIA and interaction with the PVA chains result in higher stored energy during deformation. In contrast, the increased loss modulus in the reinforced materials may be due to higher internal friction of the PVA chains when shear stress is imposed, resulting from the possible interaction of the nanofibers with several of these chains simultaneously through hydrogen bonding [79,81].

As expected, increased crosslinking density, reported to occur during gamma irradiation of PVA hydrogels, also resulted in higher storage moduli (up to 1.9 times). These results are in agreement with previous observations from other authors [81], who found that PVA hydrogels showed increasing storage moduli with gamma irradiation doses up to 30 kGy. In this case, higher doses led to more radicals and chemical bonds within the polymer matrix, with subsequent formation of denser networks and increased moduli.

It is worth highlighting that the complex shear modulus (G*) of the produced reinforced hydrogels, given by Equation (1),

(1) $$G^{*}=\sqrt{G^{\prime }+G^{\prime \prime }},$$

is within the range reported for articular cartilage (0.2–2.5 MPa) [48]. In particular, the irradiated hydrogel PVA10/PMIA1.5_G showed by far the best rheological performance, with a G* of around 0.48 MPa, comparable to the most widely reported shear modulus of natural cartilage (0.5 MPa [82,83]).

### 2.6. Tribological Behavior

**Figure 7:**
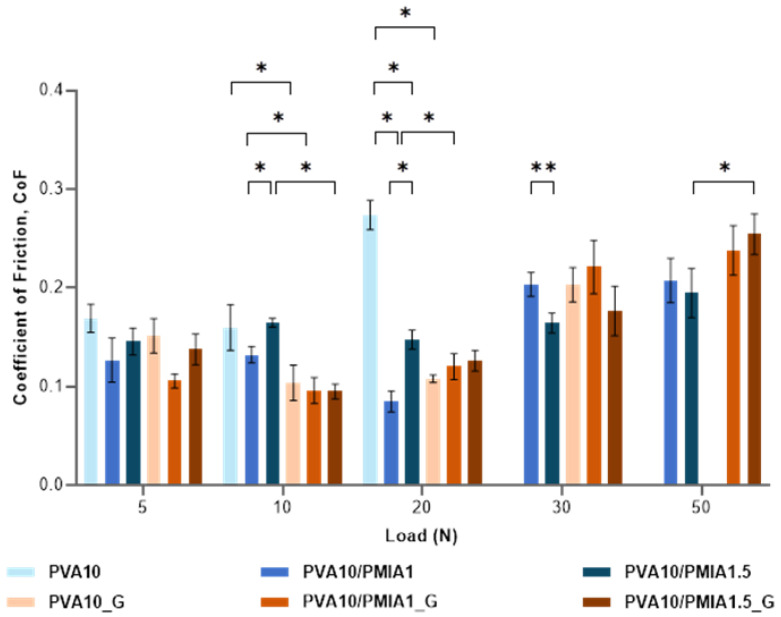
Friction coefficients of PVA- and PMIA-reinforced hydrogels before and after sterilization were measured against stainless steel 316L in PBS solution. Data followed a normal distribution: Welch’s ANOVA and Dunnett’s C test or Student’s *t* test. *p* ≤ 0.05 (*), *p* ≤ 0.01 (**).

The study of the frictional properties of materials intended for the repair of articular cartilage is essential, given the function of cartilage to provide a low-friction load-bearing interface between two articulating condyles. As such, to investigate the impact of the incorporation of the PMIA reinforcement and the sterilization of the hydrogels, the coefficient of friction (CoF) of the samples was determined by reciprocating linear motion against a 316L SS ball in a PBS solution under 5–50 N loads. Figure 7 illustrates the obtained results for the different applied loads.

When the load is increased from 5 to 10 N, in general, it is observed a decrease in the CoF; however, above these loads, the increase in the normal force causes an increase in the CoF. The same behavior is observed in articular cartilage [84]. The low CoF of hydrogels at low loads is generally assumed to be mainly linked to water’s hydrodynamic lubrication and fluid load support. The increase in the applied load translates into higher pressure that causes the squeezing of more water from the hydrogel matrix to the friction interface. The exuded fluid enhances the lubrication between the hydrogel and its counterpart, thereby leading to the decrease in the CoF [85,86,87]. On the other hand, as the load is increased, a greater hydrogel deformation occurs, increasing the contact area between the sliding surfaces, which results in a higher CoF [24,70]. In the present case, for loads above 20 N, this latter effect prevails. Chen et al. [87] also observed an increase in the friction coefficient with the contact load, which is related to the decrease in the fluid load support.

It was found that PVA10 did not withstand loads above 20 N, likely due to its lower mechanical performance, whereas the sterilization of this sample allowed it to withstand loads up to 30 N. Only the samples reinforced with PMIA could withstand the highest applied normal load (50 N). This behavior can be attributed to the superior mechanical performance of these samples.

The friction coefficients obtained for the PVA control sample, PVA10, were between 0.16 and 0.27. Although numerous authors have reported the CoF of other PVA hydrogels, a direct comparison with the published values is not straightforward, given that there are no standardized conditions. In fact, the CoF values are significantly influenced by the system geometry, counterface, testing parameters (e.g., applied load, sliding speed), and lubricating conditions, among other factors [44,66], so most results are not comparable. This is why, in the literature, the CoF values of PVA hydrogels vary significantly. Oliveira et al. [44] reported CoFs between 0.062 and 0.115 for freeze-thawed and cast-dried PVA hydrogels under a 10 N normal load against a stainless steel (SS) ball, determined using a pin-on-disc tribometer in reciprocal oscillating mode, lubricated with PBS. On the other hand, Shi et al. [69] tested freeze-thawed hydrogels against a CoCrMo ball in deionized water under 5 N using reciprocal friction mode and obtained a CoF of 0.29.

The obtained results show that, in general, the reinforcement and the sterilization of the hydrogels did not impact the CoF values much. The differences between the samples were only statistically significant in certain conditions, such as under a normal load of 10 N and 20 N. For low loads, the incorporation of the nanofibers and the exposure to gamma radiation tended to decrease the CoF of the samples. This can be attributed to the high load-bearing capacity of these materials, which diminishes the contact area during sliding, causing a reduction in friction [69].

The friction coefficient values of articular cartilage reported in the literature vary widely, from as low as 0.001 to as high as 0.46 [84,88,89]. Considering that the hydrogels produced here all showed CoF values between 0.085 and 0.274, it can be assumed that they demonstrate adequate frictional properties for the repair of articular cartilage. Still, according to the Hertzian contact theory, the normal contact loads applied in these tests (5–50 N) give rise to maximum contact pressures of 0.6–1.2 MPa, which, depending on the joint location, are within the typical stresses exerted on cartilage, usually around 0.1–5 MPa [90]. This must be kept in mind when considering the potential use of these materials, especially those that could not withstand the highest normal force tested.

As mentioned, the above CoF values of the hydrogels shown in Figure 6 were obtained against an SS ball in PBS as it allows for consistent and reproducible testing conditions, allowing the comparison between materials. Additionally, SS is widely used as a counter body for tribological testing of hydrogels and articular cartilage. However, it should be noted that these conditions are harsher than the physiological, considering that SS is much harder than cartilage, and PBS does not contain the synovial fluid (SF) components (e.g., hyaluronan, lubricin, proteoglycan 4 (PRG4), and phospholipids) mainly responsible for boundary lubrication.

**Figure 8:**
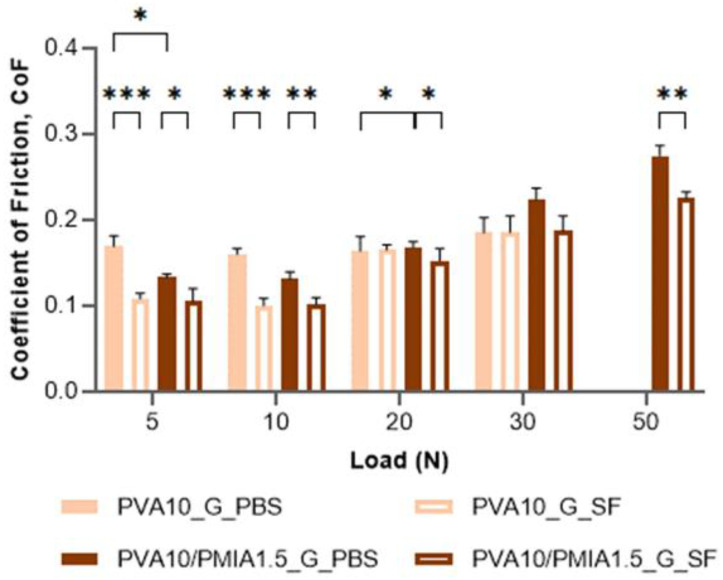
Friction coefficients of PVA10_G and PVA10/PMIA1.5_G were measured against porcine cartilage using PBS solution and SF fluid as lubricants. Data followed a normal distribution: Student’s *t* test, *p* ≤ 0.05 (*), *p* ≤ 0.01 (**), *p* ≤ 0.001 (***).

Therefore, the tribological performance of PVA10/PMIA1.5_G was investigated under more biomimetic conditions (against a porcine cartilage pin, using PBS and human SF as lubricants). This hydrogel was chosen due to its significantly superior mechanical performance compared to the remaining hydrogels. The obtained CoF values are given in Figure 8. As can be seen, there was again no consistent difference between the reinforced material and the control (PVA10_G). Additionally, even with the increased contact area between the sliding surfaces (in this case, a pin-shaped osteochondral plug with a diameter of 6 mm was used instead of a sphere), the CoF values (between 0.1 and 0.275) were still within the range of the values reported in the literature for cartilage [88,90,91].

When SF is employed instead of PBS, there is generally a significant decrease in the CoF, with an average of 18%. This was expected, considering that the viscosity (at 1 Hz shear rate) of synovial fluid is almost ten times higher than that of PBS. This increased viscosity should lead to a thicker lubricating layer between the contact surfaces. This enhances the hydrodynamic component of lubrication, reducing the friction between the sliding surfaces [46,92]. Moreover, the various biomolecules in SF contribute to the boundary lubrication of opposing cartilage tissue tissues. As such, these molecules adsorb onto the cartilage surface under high loads or low sliding speeds, creating a sacrificial layer that protects cartilage from friction [84].

### 2.7. Proof of Concept Using a Hip Simulator

The use of joint simulators before animal models is essential for ethical, scientific, and practical reasons. They provide a controlled, reproducible, and cost-effective means to evaluate the safety, feasibility, and performance of materials, minimizing the need for animal testing and ensuring that only the most promising ones proceed to the more complex and costly stages of animal and human trials [26,91,93]. To test the possibility of using PVA10/PMIA1.5_G hydrogel to repair cartilage defects, we designed a proof-of-concept experiment where hydrogel plugs were inserted in porcine femoral heads and tested against the correspondent acetabular cups using a hip simulator.

**Figure 9:**
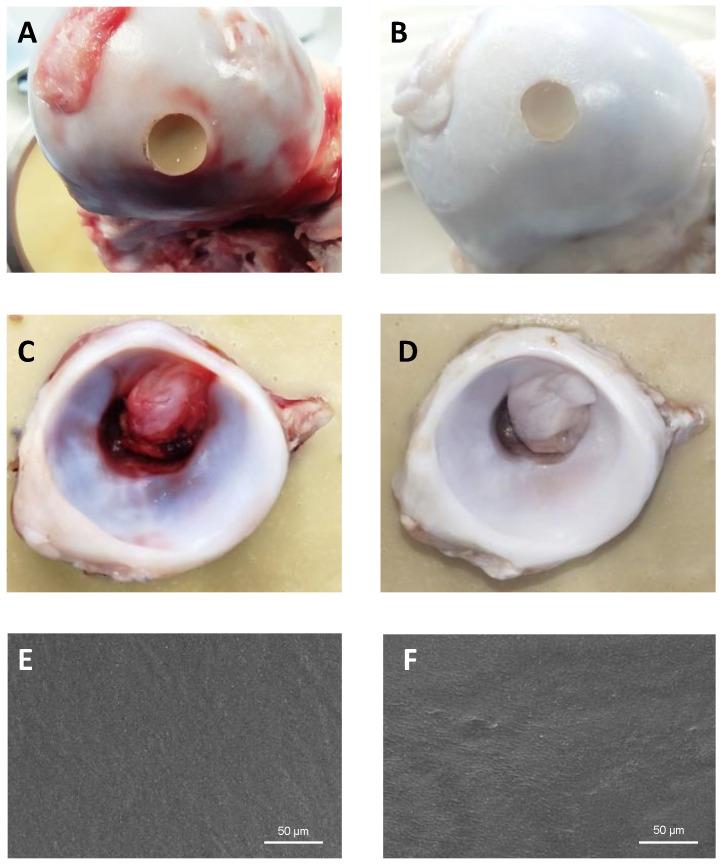
Acetabular cup (**A**,**B**) and femoral head with PVA10/PMIA1.5_G hydrogel plug (**C**,**D**) before (**A**,**C**) and after (**B**,**D**) the tests in a hip movement simulator. SEM images of the hydrogel before (**E**) and after (**F**) the simulation test.

Figure 9 presents the images of the femoral heads with the inserted hydrogel plugs and the acetabular cups before (Figure 9A,C) and after testing (Figure 9B,D) for 24 h. Visual inspection of the acetabulum showed minor roughening on the area in contact with the hydrogel. SEM images (Figure 9E,F) did not reveal any signs of wear or delamination of the hydrogel after the simulation. Only slight changes in the surface morphology were observed. In particular, PMIA particles became less visible although they remain embedded in the material, probably due to some deformation of the PVA matrix that better accommodated them. Although the outcome of the simulation is promising, further research and analysis are necessary to assess the long-term performance of the current approach. It should be noted that the simulation time was limited due to the degradation of natural tissue. However, the number of cycles can be still increased. Additionally, it would be important to investigate the use of more complex aqueous solutions to mimic the lubricant found in the joints.

### 2.8. Cytotoxicity

As for all biomaterials, it is essential to ensure that hydrogels for cartilage repair are biocompatible in order to prevent damage to the surrounding tissues upon implantation [16]. Numerous reports have demonstrated that PVA hydrogels fulfil this requisite [19,24,46,65,94]. However, such information is not available for PMIA (Nomex^®^). Still, some authors have reported the good cytocompatibility of other para-aramid nanofibers, namely those produced using Kevlar^®^ [29,35,45].

**Figure 10:**
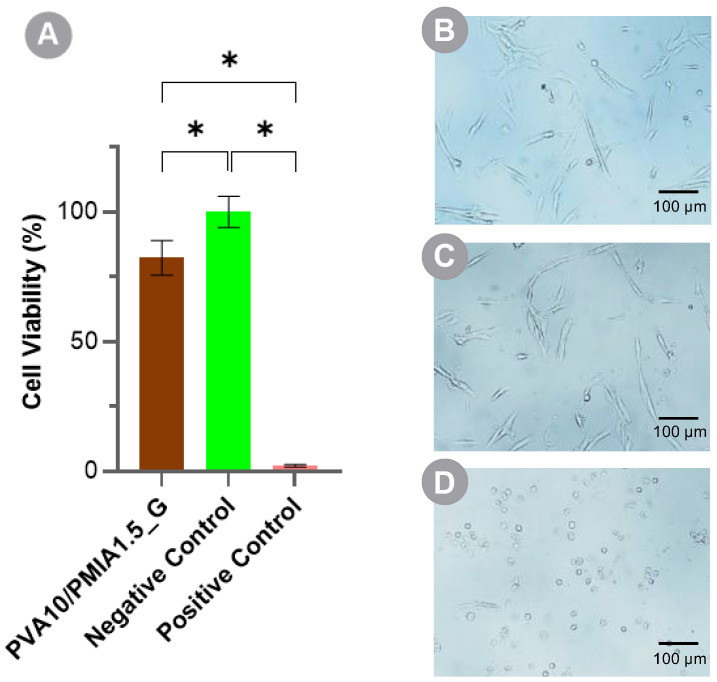
Human chondrocyte cell viability after 24 h exposure to extracts of hydrogel: MTT assay (**A**), cell morphology of PVA10/PMIA1.5_G (**B**), the negative control (**C**), and positive control (**D**). For the negative control, the cells were cultured in DMEM/F12, while for the positive control, they were cultured in DMEM/F12 with 10% (*v*/*v*) DMSO. Data followed a normal distribution: Welch’s ANOVA and Dunnett’s C test, *p* ≤ 0.05 (*).

The in vitro cytotoxicity of PVA10/PMIA1.5_G, the best performing material, was assessed using human chondrocytes (HCs). Cell viability was evaluated following a 24 h exposure to material extracts through the MTT assay. The percentage of viable chondrocytes, after incubation with control solutions (DMEM/F12 and DMEM/F12 with DMSO at 10% *v*/*v* as negative and positive controls, respectively) and the leach-out of the hydrogel, is given in Figure 10, alongside the corresponding cell morphology. The optical microscopy images of the chondrocytes reveal the proper morphology of HC cells, which present a spindle shape and high cell spread and adhesion, both for the negative control and the sample tested. On the other hand, the positive control showed most cells with a rounded morphology not adhered to the well surface, suggesting a significantly higher cell death. Although the exposure of the chondrocytes to the extracts of the hydrogel has led to a significant reduction in the cell viability compared to the negative control (82% versus 100%), it exceeded the 70% threshold [95]. Thus, it can be concluded that the hydrogel does not possess a cytotoxic effect.

## 3. Conclusions

This work aimed to develop a PVA-based composite hydrogel capable of substituting hyaline cartilage damaged due to several joint pathologies. To mimic cartilage and its function, PMIA fibers were firstly chemically treated to obtain a dispersion of spherical particles of 40–300 nm which was then blended with a PVA solution to produce reinforced hydrogels with different contents of PMIA (1 and 1.5%). The obtained hydrogels presented a compact, non-porous, and uniform microstructure. No differences in the hydrogel’s chemical structure were observed by FTIR between pristine and reinforced PVA. The interaction between the polymers occurs by hydrogen bonding, as reported for other aramid nanofiber-reinforced PVA materials. The hydrogels were further gamma irradiated to investigate the effect of a possible sterilization method on the materials’ properties. FTIR and TGA suggest that irradiation is likely to increase the crosslinking degree of the PVA network through the establishment of new hydrogen bonds.

The friction coefficient of the hydrogels against both SS and porcine cartilage did not change significantly upon reinforcement and/or gamma irradiation. However, contrary to pristine PVA, the composites were able to withstand all the tested loads (10–50 N) without suffering catastrophic failure. The hydrogel’s static and dynamic mechanical properties were significantly improved owing to the PMIA reinforcement and sterilization. In fact, both led to an increase in the compressive tangent modulus, tensile elongation at break, toughness, ultimate tensile strength, and shear modulus of the samples.

The hydrogel reinforced with 1.5% PMIA and irradiated (PVA10/PMIA1.5_G) presented an equilibrium water content and a wettability within the range found for hyaline cartilage but a superior mechanical performance. Therefore, it was selected to be tested in a hip movement simulator. Hydrogel plugs inserted in porcine femoral heads were able to withstand 66,600 cycles (corresponding to ≈24 days of walking) under a load of ≈900 N, without suffering any damage or causing significant changes in the opponent cartilage surface. Finally, cell viability tests using a human chondrocyte cell line demonstrated its non-cytotoxic character.

Overall, the study successfully met its objectives and confirmed the initial hypothesis that both the PMIA reinforcement and the gamma irradiation led to an increase in the hydrogel’s tribomechanical performance. These types of materials present significant clinical implications since they are expected to enhance joint function and lifespan and reduce pain, which will improve the patient’s quality of life. A better tribomechanical behavior will reduce the need for revision surgeries, thereby improving patient outcomes and reducing healthcare costs.

## 4. Materials and Methods

### 4.1. Materials

Poly (m-phenylene isophthalamide) (PMIA) Nomex^®^ fibers (Sewfil Daflame 40) were bought from iDovy (Yecla, Spain). Poly(vinyl alcohol) powder (PVA, Mw 146–186 kDa, hydrolyation degree ≥ 99%), phosphate buffer saline (PBS), hydrochloric acid 37% (HCl, ACS reagent), isopropanol, penicillin–streptomycin, fetal bovine serum (FBS), and 3-(4,5-dimethylthiazol-2-yl)-2,5-diphenyltetrazolium bromide (MTT) were obtained from Sigma-Aldrich (St. Louis, MO, USA). Potassium hydroxide (KOH, purity > 85%) and Dimethyl sulfoxide (DMSO, purity ≥ 99.9%) were supplied by ChemLab NV (Zedelgem, Belgium) and Fisher Scientific^TM^ (Loughborough, UK), respectively. A Millipore system was used to obtain ultrapure water (18 MΩ cm). Polyamide/polyethylene bags were procured from Penta Ibérica (Torres Vedras, Portugal). Sodium hydroxide (NaOH, ≥99%) was obtained from Merck (Darmstadt, Germany), while sodium chloride (NaCl, ≥99%) was from PanReac AppliChem (Darmstadt, Germany. Dulbecco’s Modified Eagle’s Medium/Nutrient Mixture F-12 Ham (mixture 1:1) with glutamine (DMEM/F12) was obtained from Gibco^TM^, Thermo Fisher (Waltham, MA, USA). Octylphenoxy poly(ethyleneoxy)ethanol (IGEPAL^®^) was from Merck KGaA (Darmstadt, Germany). The components for the preparation of the acrylic resin used for the simulation tests, Centri™ Tooth Shades Cold Cure Powder and Centri™ Base CC Liquid, were obtained from WhW (Hull, UK).

### 4.2. Preparation of the Materials

PMIA (chemical structure in Figure 1) fibers were treated with a mixture of DMSO and KOH, adapting a previously reported methodology [29,33,36]. In this case, 8% *w*/*v* of chopped PMIA fibers were added to a mixture of 3% *w*/*v* KOH in DMSO and left under magnetic stirring at room temperature for at least 12 days. This formed a uniform dispersion of PMIA, which was diluted with DMSO to produce solutions of different concentrations (4% and 6% *w*/*v*).

**Figure 11:**
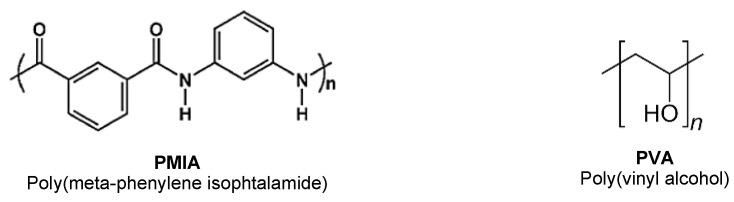
Chemical structure of poly(meta-phenylene isophthalamide (PMIA) and poly(vinyl alcohol) (PVA).

Meanwhile, a PVA (chemical structure in Figure 11) solution was prepared in DMSO at 13.33% *w*/*v*. The dissolution took place at 90 °C and was complete within 20 h. During this process, the mixture was manually stirred every hour for the first 6 h to ensure homogeneity.

Once dissolution was complete, the PVA solution and the PMIA nanofiber dispersion were blended at a ratio of 3:1 (*v*/*v*) to obtain PVA10/PMIA1 and PVA10/PMIA1.5, containing 10% wt. of PVA and 1% and 1.5% wt. of PMIA, respectively. The combined solutions were vigorously shaken for approximately 15 s and promptly immersed in a water bath set to 90 °C under magnetic stirring for 2 h. The mixture was then placed in an ultrasonic bath for another 2 h at 80 °C to remove all air bubbles. Thereafter, it was poured into pre-heated (at 90 °C) glass Petri dishes (80 mm diameter) that were covered and left at room temperature for 12 h. This allowed the mixtures to cool and the gelation process to begin. After this period, the lids were taken off, and the gels were put in a fume hood for a further 12 h to guarantee full gelation. The resulting materials (4 mm thick) were then immersed in 0.5 L of ultrapure water for 3 to 5 days. This was replaced three times a day to exchange the solvent. Then, they underwent drying at 37 °C in an oven with air circulation for 24 h, followed by 24 h more under vacuum to ensure complete removal of solvents. Before each characterization test, the hydrogels were rehydrated in ultrapure water until equilibrium, for at least 48 h, and cut with punchers with the appropriate dimensions. For the sake of comparison, pristine PVA hydrogels (PVA10) were also prepared by diluting in a ratio of 3:1 (*v*/*v*) the PVA solution in pure DMSO.

### 4.3. Sterilization

The samples were sterilized by packing the materials in sealed polyamide/polyethylene bags, in oxygen-free ultrapure water, and deaerated by bubbling nitrogen gas through water for at least 30 min. For cytotoxicity tests, discs with 6 mm diameter were previously cut. The packed samples were gamma irradiated with a dose of 25 kGy (5 kGy/h) using a ^60^Co source at room temperature. The absorbed dose was confirmed using Red 4304 dosimeters (Harwell Dosimeters, Didcot, Oxfordshire, UK). The sterilized samples were designated PVA10_G, PVA10/PMIA1_G, and PVA10/PMIA1.5_G.

### 4.4. Morphological Characterization

AFM (Easyscan2, Nanosurf, Liestal, Switzerland) was used to assess the morphology of PMIA fibers after treatment. Samples were prepared by dipping a clean glass slide into a PMIA diluted dispersion (100×) and rinsing it with water, followed by air drying. Images of 5 × 5 mm^2^ (n ≥ 3) were obtained in tapping mode using a silicon probe with an applied voltage of 300 mV and free vibration amplitude of 96 mV at room temperature (20 °C).

The morphology of the hydrogels’ surface and cross-cut sections was observed by SEM (Phemon ProX, Thermo Fisher Scientific, Cambridge, UK) using an acceleration voltage of 10 kV. The hydrated samples (disks with a diameter of 12 mm) were immersed in N_2_ for 10 s and then fractured. After, they were lyophilized for 24 h in a freeze dryer (Alpha 1-2 LDplus, Martin Christ, Osterode, Germany). Before SEM analysis, a conductive layer of Au:Pd (20:80) was applied over the samples’ surface using a turbo-pumped sputter coater (Q150T ES, Quorum Technologies, Lewes, UK). EDS (Bruker, Coventry, UK) coupled to SEM equipment was used for chemical analysis.

### 4.5. Chemical and Thermal Characterization

FTIR analyses were carried out with Spectrum Two TM (PerkinElmer, Waltham, MA, USA) equipment with a PerkinElmer Universal Attenuated Total Reflectance (UATR) Two Accessory to investigate the chemical structure of the materials. Before measurements, discs with 6 mm diameter (n ≥ 3) were placed for 48 h at 37 °C in a vacuum oven to eliminate residual water. The spectra were acquired in the range 4000–400 cm^−1^, with 4 cm^−1^ resolution, and by averaging eight scans. All obtained spectra were normalized.

TGA was performed using a STA 7200 Thermal Analysis System (Hitachi, Ibaraki, Japan). The analysis was conducted over a temperature range of 40 to 600 °C, with a rate of heating/cooling of 10 °C/min and with nitrogen gas purging (50 mL/min), n ≥ 3.

### 4.6. Equilibrium Water Content and Wettability

Hydrated sample discs (diameter 10 mm, n = 5) were gently blotted with lab paper to remove the water at the surface and weighed using a semi-micro analytical balance (Discovery DV215CD, Ohaus Corporation, Parsippany, NJ, USA). Then, they were dried at 37 °C until constant weight. To determine the equilibrium water content (EWC), Equation (2) was used:

(2) $$EWC\;(\%)=100\times (W_{h}-W_{d})/W_{h},$$

where *W_h_* is the weight of the hydrated sample and *W_d_* of the dried sample.

The captive bubble method was used to study the wettability of the hydrogels (n = 5). A metallic support was used to fix the hydrated samples and then, it was placed facing downward in a liquid cell with ultrapure water. A micrometric syringe with a hook-shaped needle was used to produce air bubbles (≈4 μL) on the hydrogel surface. After bubble deposition, images were captured at predetermined times for 30 s using a video camera (jAi CV-A50, Spain), connected to an optical microscope (Wild M3Z, Leica Microsystems, Wetzlar, Germany), and coupled to a frame grabber (DT3155, Data Translation, Norton, Tempe, AZ, USA). The contact angle was determined at room temperature using the ADSA-P (Axisymmetric Drop Shape Analysis-Profile, Applied Surface Thermodynamics Research Associates, Toronto, ON, Canada) software.

### 4.7. Mechanical Properties

Compressive tests were performed in unconfined mode in ultrapure water using the same equipment as the tensile tests at room temperature. At the beginning of each test, 2 N was applied as preload. Hydrated discs (8 mm diameter, n ≥ 3) of each material were compressed at a speed of 0.1 mm/s until reaching 5 kg. The same speed was set for unloading. For each hydrogel, the compressive tangent modulus ($E_{\varepsilon }$) was determined between 5 and 35% strain, in increments of 5%, following Equation (3) [46]:

(3) $$E_{\varepsilon }={(\sigma }_{\varepsilon +∆\varepsilon }-\sigma_{\varepsilon -∆\varepsilon })/2∆\varepsilon ,$$

where the difference of strain value ∆ε was 1%. 

Tensile tests were carried out using a TA.XT Express Texture Analyser (Stable Micro Systems, Godalming, UK). The tests were conducted at a speed of 0.5 mm/s until failure at room temperature using hydrated dumbbell-shaped samples (2.5 mm width, 10 mm gauge length, n ≥ 3). The elastic modulus, elongation to break, and ultimate tensile strength (UTS) were determined from the stress–strain curves.

### 4.8. Rheological Properties

Rheological measurements were performed using a Modular Compact Rheometer (MCR-92, Anton Paar, Graz, Austria) at 37 °C with a 25 mm diameter parallel measuring plate (PP25). Before the tests, the hydrated discs (25 mm diameter, n ≥ 3) were compressed by 2% for 15 min with water surrounding them. Firstly, to identify the linear viscoelastic region, amplitude sweeps were performed over a shear strain range of 0.01–10% with an angular frequency of 1 Hz. With a fixed strain amplitude of 0.1%, the hydrogels underwent oscillatory frequency sweep tests across a range of 0.1–10 Hz, which allowed us to obtain the storage (G′) and loss (G″) moduli.

### 4.9. Tribological Behavior

The tribological behavior of the produced hydrogels against either 6 mm diameter 316L stainless steel (SS) balls (Luis Aparicio SL, Barcelona, Spain) or porcine articular cartilage pins was assessed using a pin-on-disc tribometer (TRB3, Anton Paar, Graz, Austria) (n = 3). The hydrated hydrogels were pre-equilibrated in the lubricating media: PBS solution or human synovial fluid (SF).

Reciprocating linear sliding tests (1200 cycles) were carried out with a sliding distance of 8 mm, frequency of 1 Hz (representing normal walking conditions), and normal contact loads of 5 N and 50 N at room temperature.

Regarding the porcine cartilage specimens, freshly retrieved adult porcine femurs were acquired from a slaughterhouse. Pins were taken from the flat areas of the porcine femoral condyles using a hollow drill. The osteochondral plugs (~6 mm in diameter, ~4 mm in thickness) were washed with ultrapure water to remove debris before being placed in PBS solution and stored at −20 °C until the experiments were performed.

### 4.10. Hip Movement Simulation

Tribological performance and proof of concept of the best-performing hydrogel were further assessed with porcine hip joint with the use of a hip simulator, whereby a hydrogel plug was inserted into a porcine femoral head and articulated against the paired porcine acetabulum.

#### 4.10.1. Preparation and Fixation of the Hip Joints

Porcine hip joints were prepared for anatomical hip simulation following the procedure described previously by Jimenez-Cruz et al. [96]. In brief, this consisted of dissection and fixturing to ensure the joint was positioned at an anatomically relevant angle in the simulator. Hind legs were sourced from a slaughterhouse (John Penny & Son’s, Leeds, UK) within 24–48 h of slaughter. The joints were disarticulated by carefully removing the capsule and ligament teres. The cartilage surfaces were kept hydrated throughout the sample preparation and fixation by regularly spraying it with PBS. The femoral heads and acetabula were mounted using acrylic-based resin in custom-made fixtures. The acetabular cup was aligned at 30°, measured between the sagittal plane and a plane passing through the center of the transverse acetabular ligament (TAL). Additionally, the inclination of the acetabulum was set at 35°, measured between the acetabular rim and the transverse plane. The femur was anatomically positioned using a customized potting fixture and cemented into a testing cup.

#### 4.10.2. Hydrogel Plug Preparation

The best-performing hydrogel (PVA10/PMIA1.5_G) was inserted as a plug in the femoral heads. First, osteochondral grafts were harvested from a donor femoral head. Cylindrical bone plugs with cartilage on the top (6.5 mm diameter and 15 mm depth) were extracted from a donor femoral head (Figure S5A,B). Then, the cartilage was meticulously removed (Figure S5C). A sample of the hydrogel with the same diameter was fixed on the top of each bone plug in a precise manner with glue (Loctite^®^ super glue-3) (Figure S5D). The implantation of the graft plugs in the recipient femurs was achieved by drilling a cavity (6.5 mm diameter and 12 mm depth) at the posterior–superior area. The cavity was irrigated to eliminate debris; then, the depth was measured to correct the graft plug height. The graft plug with the hydrogel was shortened to match the cavity dimension. A dilator cylinder was used to smooth the borders and avoid excessive pressure while inserting the plug. The plug was gently inserted in the cavity, verifying that the graft remained attached at the same surrounding cartilage height with no more than 0.5 mm protuberant or depression difference (Figure S5E).

#### 4.10.3. Experimental Testing: Setup and Parameters

An anatomical hip simulator (Anatomical Hip Simulator, ProSim, Manchester, UK) was used to conduct ex vivo experimental simulations and provide evidence in terms of the proof of concept. This simulator allows the application of flexion–extension (FE), interior–exterior (IE), and abduction–adduction (AA) motions. A silicon gaiter was used to encapsulate the fixed joint that was immersed in PBS to maintain proper lubrication during simulation.

Loading and motion profiles are based on ISO 14242-1:2014 [97] total hip replacement (THR) wear testing modified for porcine tissue. The axial load profile was appropriately scaled to account for the differences in loading experienced by quadrupeds. As a result, the peak load applied during the testing was set at ≈900 N. Simultaneous movements mirroring human gait were applied to the femoral head as follows: −14.3° to 20.0° of FE to −4.5° to 8.2° of AA and −9.8° to 1.7° of IE rotation. Each test (n = 2) was conducted at a frequency of 1 Hz, simulating a total of 66,600 cycles, equivalent to 24.3 days of continuous walking.

To evaluate tissue conditions and document the location and extent of damage in both the articular cartilage and hydrogel, photographs were captured using a Canon 750D camera before and after the simulation. The hydrogels were retrieved from the femoral heads, washed with ultrapure water, lyophilized, and observed by SEM, following the outlined procedure in Section 4.4.

### 4.11. Cytotoxicity

Human chondrocyte (HC) cells were carefully seeded in 96-well plates (1 × 10^4^ cells/well) and cultured in DMEM/F12 supplemented media at 37 °C with a humidified atmosphere containing 5% CO_2_. After 24 h, the culture medium was replaced by hydrogel extracts containing eventual leached-out products, and the cells were incubated for 24 h more under the aforementioned conditions. To prepare the extracts, the hydrogel samples were sterilized in water by gamma irradiation, as described in Section 4.3. The extracts were obtained according to ISO 10993-12 guidelines. Briefly, discs (6 mm in diameter) of the selected hydrogels were incubated in culture medium, in closed containers, at a ratio of 3 cm^2^/mL (samples’ area/volume of medium) for 24 h at 37 °C in a humidified atmosphere containing 5% CO_2_. Positive and negative controls were obtained by replacing the culture medium with 10% (*v*/*v*) DMSO culture medium and fresh cell culture medium, respectively. The experiments were conducted in accordance with ISO 10993-5:2009 [95] and ISO 10993-5:2012(E) [98].

After the 24 h incubation period, cell viability was quantified through an MTT assay (n = 5). In this case, culture medium present in each well was replaced by 50 μL of 0.5 mg/mL MTT solution in serum-free DMEM medium. After 3 h incubation at 37 °C and 5% CO_2_, the diluted MTT solution was carefully removed, and 150 μL of MTT solvent (4 mM HCl and 0.1% (*v*/*v*) IGEPAL in isopropanol) was added to each well. Then, the MTT formazan crystals were dissolved, and the absorbance of the samples was measured at 595 nm using a microplate reader (Infiniti 200 Pro, Tecan, Männedorf, Switzerland). The cell viability percentage was determined by normalizing the results with the negative control (100% cell viability).

### 4.12. Statistical Analysis

Statistical analysis was performed using IBM^®^ SPSS^®^ Statistics software (v.26) from IBM Corporation, Armonk, NY, USA. Quantitative results are given as mean ± standard deviation. The data distribution normality was assessed using the Shapiro–Wilk test. When they followed a normal distribution, Levene’s test was applied to evaluate the similarity of variances between groups. When variances were found to be similar, one-way ANOVA was performed followed by Tukey’s test for multiple comparisons. In cases where the assumption of equal variances was not verified, Welch’s ANOVA and Dunnett’s C test were employed to identify different pairs of groups. Student’s *t* test was used for pairwise comparisons when necessary. Non-parametric tests were performed for data not following a normal distribution. Kruskal–Wallis tests adjusted with Bonferroni correction were used to determine significant differences between 3 groups, while Mann–Whitney U tests were applied for comparisons between 2 groups. The level of significance was 0.05 for all tests. Asterisks (*) were used to denote statistically significant differences between groups as follows: *p* ≤ 0.05 (*), *p* ≤ 0.01 (**), and *p* ≤ 0.00 1 (***).

## Data Availability

The raw data supporting the conclusions of this article will be made available by the author on request: carolina.marto.costa@tecnico.ulisboa.pt.

## Supplementary Materials

The following supporting information can be downloaded at: https://www.mdpi.com/article/10.3390/gels10080514/s1, Figure S1. AFM image of the PMIA nanoparticles; Figure S2. EDS analysis of the spherical structures within the reinforced hydrogels (left) compared to the surrounding material (right). The data presented are from the analysis of the sample PVA10/PMIA1.5_G; Figure S3. ATR-FTIR spectra of commercial PMIA fibers, commercial PVA powder, and hydrogels PVA10 and PVA10/PMIA1.5 before and after sterilization; Figure S4. (A) TGA curves of irradiated and non-irradiated PVA10 and PVA10/PMIA1.5. (B) Temperature at which the samples loss 10% weight (T0.1).; Figure S5. Schematic representation of the hydrogel-modified bone plug insertion procedure.

## References

[1] Ledingham J., Snowden N., Ide Z. (2017). Diagnosis and early management of inflammatory arthritis. BMJ.

[2] Katta J., Pawaskar S.S., Jin Z.M., Ingham E., Fisher J. (2007). Effect of load variation on the friction properties of articular cartilage. Proc. Inst. Mech. Eng. Part J J. Eng. Tribol..

[3] Danso E., Honkanen J., Saarakkala S., Korhonen R. (2014). Comparison of nonlinear mechanical properties of bovine articular cartilage and meniscus. J. Biomech..

[4] Murakami T., Yarimitsu S., Sakai N., Nakashima K., Yamaguchi T., Sawae Y., Suzuki A. (2017). Superior lubrication mechanism in poly(vinyl alcohol) hybrid gel as artificial cartilage. Proc. Inst. Mech. Eng. Part J J. Eng. Tribol..

[5] Katz J.N., Arant K.R., Loeser R.F. (2021). Diagnosis and Treatment of Hip and Knee Osteoarthritis: A Review. JAMA.

[6] Cross M., Smith E., Hoy D., Nolte S., Ackerman I., Fransen M., Bridgett L., Williams S., Guillemin F., Hill C.L. (2014). The global burden of hip and knee osteoarthritis: Estimates from the Global Burden of Disease 2010 study. Ann. Rheum. Dis..

[7] Beddoes C.M., Whitehouse M.R., Briscoe W.H., Su B. (2016). Hydrogels as a Replacement Material for Damaged Articular Hyaline Cartilage. Materials.

[8] Jeuken R.M., van Hugten P.P., Roth A.K., Timur U.T., Boymans T.A., van Rhijn L.W., Bugbee W.D., Emans P.J. (2021). A Systematic Review of Focal Cartilage Defect Treatments in Middle-Aged Versus Younger Patients. Orthop. J. Sports Med..

[9] Gowd A.K., Weimer A.E., Rider D.E., Beck E.C., Agarwalla A., O’brien L.K., Alaia M.J., Ferguson C.M., Waterman B.R. (2021). Cartilage Restoration for Tibiofemoral Bipolar Lesions Results in Promising Failure Rates: A Systematic Review. Arthrosc. Sports Med. Rehabil..

[10] Orthopaedics J.R. MaioRegen Ostheochondral Substitute. https://jri-ltd.com/our-products/orthobiologics/maioregen.

[11] Stryker Cartiva Synthetic Cartilage Implant. https://www.stryker.com/us/en/foot-and-ankle/products/cartiva.html.

[12] BioPoly. https://biopolyortho.com/.

[13] Brix M., Kaipel M., Kellner R., Schreiner M., Apprich S., Boszotta H., Windhager R., Domayer S., Trattnig S. (2016). Successful osteoconduction but limited cartilage tissue quality following osteochondral repair by a cell-free multilayered nano-composite scaffold at the knee. Int. Orthop. (SICOT).

[14] Radcliffe M.J., Roukis T.S. (2024). A review of adverse events related to synthetic cartilage implant for the first metatarsophalangeal joint: An analysis of Manufacturer and User Facility Device Experience database from 2016 to 2023. Foot Ankle Surg. Tech. Rep. Cases.

[15] Oliveira A.S., Silva J.C., Loureiro M.V., Marques A.C., Kotov N.A., Colaço R., Serro A.P. (2023). Super-Strong Hydrogel Composites Reinforced with PBO Nanofibers for Cartilage Replacement. Macromol. Biosci..

[16] Zhu L., Liu Y., Jiang Z., Sakai E., Qiu J., Zhu P. (2019). Highly temperature resistant cellulose nanofiber/polyvinyl alcohol hydrogel using aldehyde cellulose nanofiber as cross-linker. Cellulose.

[17] Chronopoulou L., Cacciotti I., Amalfitano A., Di Nitto A., D’arienzo V., Nocca G., Palocci C. (2021). Biosynthesis of innovative calcium phosphate/hydrogel composites: Physicochemical and biological characterisation. Nanotechnology.

[18] Mahinroosta M., Farsangi Z.J., Allahverdi A., Shakoori Z. (2018). Hydrogels as intelligent materials: A brief review of synthesis, properties and applications. Mater. Today Chem..

[19] Branco A.C., Oliveira A.S., Monteiro I., Nolasco P., Silva D.C., Figueiredo-Pina C.G., Colaço R., Serro A.P. (2022). PVA-Based Hydrogels Loaded with Diclofenac for Cartilage Replacement. Gels.

[20] Li F., Wang A., Wang C. (2016). Analysis of friction between articular cartilage and polyvinyl alcohol hydrogel artificial cartilage. J. Mater. Sci. Mater. Med..

[21] Baker M.I., Walsh S.P., Schwartz Z., Boyan B.D. (2012). A review of polyvinyl alcohol and its uses in cartilage and orthopedic applications. J. Biomed. Mater. Res. Part B Appl. Biomater..

[22] Nečas D., Yarimitsu S., Rebenda D., Shinmori H., Vrbka M., Sawae Y., Murakami T., Křupka I. (2023). On the replacement of articular cartilage: The friction of PVA hydrogel layer in hip simulator test. Tribol. Int..

[23] Maiolo A.S., Amado M.N., Gonzalez J.S., Alvarez V.A. (2012). Development and characterization of Poly (vinyl alcohol) based hydrogels for potential use as an articular cartilage replacement. Mater. Sci. Eng. C.

[24] Oliveira A.S., Schweizer S., Nolasco P., Barahona I., Saraiva J., Colaço R., Serro A.P. (2020). Tough and Low Friction Polyvinyl Alcohol Hydrogels Loaded with Anti-inflammatories for Cartilage Replacement. Lubricants.

[25] Zhong Y., Lin Q., Yu H., Shao L., Cui X., Pang Q., Zhu Y., Hou R. (2024). Construction methods and biomedical applications of PVA-based hydrogels. Front. Chem..

[26] Trucco D., Vannozzi L., Teblum E., Telkhozhayeva M., Nessim G.D., Affatato S., Al-Haddad H., Lisignoli G., Ricotti L. (2021). Graphene Oxide-Doped Gellan Gum–PEGDA Bilayered Hydrogel Mimicking the Mechanical and Lubrication Properties of Articular Cartilage. Adv. Healthc. Mater..

[27] Jalageri M.B., Kumar G.C.M. (2022). Hydroxyapatite Reinforced Polyvinyl Alcohol/Polyvinyl Pyrrolidone Based Hydrogel for Cartilage Replacement. Gels.

[28] Song K., Zhu W., Li X., Yu Z. (2020). A novel mechanical robust, self-healing and shape memory hydrogel based on PVA reinforced by cellulose nanocrystal. Mater. Lett..

[29] Xu L., Zhao X., Xu C., Kotov N.A. (2018). Water-Rich Biomimetic Composites with Abiotic Self-Organizing Nanofiber Network. Adv. Mater..

[30] Deopura B.L., Padaki N.V. (2015). Synthetic Textile Fibres. Textiles and Fashion.

[31] Du W., Zhang J., Zhao Z., Zhang X. (2020). Preparation of novel temperature-responsive double-network hydrogel reinforced with aramid nanofibers. Compos. Commun..

[32] Avci H., Hassanin A., Hamouda T., Kiliç A. (2019). High performance fibers: A review on current state of art and future challenges. Eskişehir Osman. Üniversitesi Mühendislik Ve Mimar. Fakültesi Derg..

[33] Yang M., Cao K., Sui L., Qi Y., Zhu J., Waas A., Arruda E.M., Kieffer J., Thouless M.D., Kotov N.A. (2011). Dispersions of Aramid Nanofibers: A New Nanoscale Building Block. ACS Nano.

[34] Guan Y., Li W., Zhang Y., Shi Z., Tan J., Wang F., Wang Y. (2017). Aramid nanofibers and poly (vinyl alcohol) nanocomposites for ideal combination of strength and toughness via hydrogen bonding interactions. Compos. Sci. Technol..

[35] Guo Y., An X., Fan Z. (2021). Aramid nanofibers reinforced polyvinyl alcohol/tannic acid hydrogel with improved mechanical and antibacterial properties for potential application as wound dressing. J. Mech. Behav. Biomed. Mater..

[36] Kotov N.A., Yang M., Cao K., Thouless M.D., Arruda E.M., Waas A.M., Siepermann C.A.P., Anderson R.M. (2012). Synthesis and Use of Aramid Nanofibers. U.S. Patent.

[37] DuPont Nomex® Fiber Technical Guide. https://www.dupont.com/content/dam/dupont/amer/us/en/personal-protection/public/documents/en/Nomex(R)%20Fiber%20Technical%20Guide.pdf.

[38] Kim S., Jeong J.-O., Lee S., Park J.-S., Gwon H.-J., Jeong S.I., Hardy J.G., Lim Y.-M., Lee J.Y. (2018). Effective gamma-ray sterilization and characterization of conductive polypyrrole biomaterials. Sci. Rep..

[39] dos Santos V., Brandalise R.N., Savaris M. (2017). Biomaterials Sterilization Methods. Engineering of Biomaterials.

[40] Sasaki S., Suzuki A. (2016). Factors influencing the swelling and elution properties of poly(vinyl alcohol) cast gels. Polym. Adv. Technol..

[41] Yang B., Wang L., Zhang M., Luo J., Ding X. (2019). Timesaving, High-Efficiency Approaches To Fabricate Aramid Nanofibers. ACS Nano.

[42] Marrella A., Lagazzo A., Dellacasa E., Pasquini C., Finocchio E., Barberis F., Pastorino L., Giannoni P., Scaglione S. (2018). 3D Porous Gelatin/PVA Hydrogel as Meniscus Substitute Using Alginate Micro-Particles as Porogens. Polymers.

[43] Coluccino L., Gottardi R., Ayadi F., Athanassiou A., Tuan R.S., Ceseracciu L. (2018). Porous Poly(vinyl alcohol)-Based Hydrogel for Knee Meniscus Functional Repair. ACS Biomater. Sci. Eng..

[44] Oliveira A.S., Seidi O., Ribeiro N., Colaço R., Serro A.P. (2019). Tribomechanical Comparison between PVA Hydrogels Obtained Using Different Processing Conditions and Human Cartilage. Materials.

[45] Oliveira A.S., Silva J.C., Figueiredo L., Ferreira F.C., Kotov N.A., Colaço R., Serro A.P. (2022). High-performance bilayer composites for the replacement of osteochondral defects. Biomater. Sci..

[46] Pires T., Oliveira A.S., Marques A.C., Salema-Oom M., Figueiredo-Pina C.G., Silva D., Serro A.P. (2022). Effects of Non-Conventional Sterilisation Methods on PBO-Reinforced PVA Hydrogels for Cartilage Replacement. Gels.

[47] Gomaa M.M., Hugenschmidt C., Dickmann M., Abdel-Hady E.E., Mohamed H.F.M., Abdel-Hamed M.O. (2018). Crosslinked PVA/SSA proton exchange membranes: Correlation between physiochemical properties and free volume determined by positron annihilation spectroscopy. Phys. Chem. Chem. Phys..

[48] Hong X., Zou L., Zhao J., Li C., Cong L. (2018). Dry-wet spinning of PVA fiber with high strength and high Young’s modulus. IOP Conf. Ser. Mater. Sci. Eng..

[49] Reguieg F., Ricci L., Bouyacoub N., Belbachir M., Bertoldo M. (2020). Thermal characterization by DSC and TGA analyses of PVA hydrogels with organic and sodium MMT. Polym. Bull..

[50] Jinisha B., Anilkumar K.M., Manoj M., Ashraf C.M., Pradeep V.S., Jayalekshmi S. (2019). Solid-state supercapacitor with impressive performance characteristics, assembled using redox-mediated gel polymer electrolyte. J. Solid State Electrochem..

[51] Wang L. (2010). Comparison and Analysis of Thermal Degradation Process of Aramid Fibers (Kevlar 49 and Nomex). J. Fiber Bioeng. Inform..

[52] Ng H.M., Saidi N.M., Omar F.S., Ramesh K., Ramesh S., Bashir S. (2018). Thermogravimetric Analysis of Polymers. Encyclopedia of Polymer Science and Technology.

[53] Radosavljević A., Spasojević J., Krstić J., Kačarević-Popović Z. (2019). Nanocomposite Hydrogels Obtained by Gamma Irradiation. Cellulose-Based Superabsorbent Hydrogels.

[54] Wang B., Kodama M., Mukataka S., Kokufuta E. (1998). On the intermolecular crosslinking of PVA chains in an aqueous solution by γ-ray irradiation. Polym. Gels Netw..

[55] Zhang S.-J., Yu H.-Q. (2004). Radiation-induced degradation of polyvinyl alcohol in aqueous solutions. Water Res..

[56] Martínez-Barrera G., del Coz-Díaz J.J., Álvarez-Rabanal F.P., Gayarre F.L., Martínez-López M., Cruz-Olivares J. (2020). Waste tire rubber particles modified by gamma radiation and their use as modifiers of concrete. Case Stud. Constr. Mater..

[57] Hargreaves G., Bowen J.J. (1973). Combined Effects of Gamma and Ultraviolet Radiation Plus Heat on Fibrous Polyamides. Text. Res. J..

[58] Zhou Q., Lyu J., Wang G., Robertson M., Qiang Z., Sun B., Ye C., Zhu M. (2021). Mechanically Strong and Multifunctional Hybrid Hydrogels with Ultrahigh Electrical Conductivity. Adv. Funct. Mater..

[59] Hsieh Y.-L., Yu B., Hartzell M.M. (1992). Liquid Wetting, Transport, and Retention Properties of Fibrous Assemblies: Part II: Water Wetting and Retention of 100% and Blended Woven Fabrics. Text. Res. J..

[60] Zhou C., Wu Q. (2011). A novel polyacrylamide nanocomposite hydrogel reinforced with natural chitosan nanofibers. Colloids Surf. B Biointerfaces.

[61] Sasaki S., Omata S., Murakami T., Nagasawa N., Taguchi M., Suzuki A. (2018). Effect of Gamma Ray Irradiation on Friction Property of Poly(vinyl alcohol) Cast-Drying on Freeze-Thawed Hybrid Gel. Gels.

[62] Hunt J.A., Chen R., Van Veen T., Bryan N. (2014). Hydrogels for tissue engineering and regenerative medicine. J. Mater. Chem. B.

[63] Cacopardo L., Guazzelli N., Nossa R., Mattei G., Ahluwalia A. (2019). Engineering hydrogel viscoelasticity. J. Mech. Behav. Biomed. Mater..

[64] Hao W., Yao X., Ke Y., Ma Y., Li F. (2013). Experimental characterization of contact angle and surface energy on aramid fibers. J. Adhes. Sci. Technol..

[65] Pohan G., Mattiassi S., Yao Y., Zaw A.M., Anderson D.E., Cutiongco M.F., Hinds M.T., Yim E.K. (2020). Effect of Ethylene Oxide Sterilization on Polyvinyl Alcohol Hydrogel Compared with Gamma Radiation. Tissue Eng. Part A.

[66] Shi Y., Xiong D., Zhang J. (2014). Effect of irradiation dose on mechanical and biotribological properties of PVA/PVP hydrogels as articular cartilage. Tribol. Int..

[67] El Salmawi K.M. (2007). Gamma Radiation-Induced Crosslinked PVA/Chitosan Blends for Wound Dressing. J. Macromol. Sci. Part A.

[68] Qiao K., Zheng Y., Guo S., Tan J., Chen X., Li J., Xu D., Wang J. (2015). Hydrophilic nanofiber of bacterial cellulose guided the changes in the micro-structure and mechanical properties of nf-BC/PVA composites hydrogels. Compos. Sci. Technol..

[69] Shi Y., Xiong D., Li J., Wang N. (2016). The water-locking and cross-linking effects of graphene oxide on the load-bearing capacity of poly(vinyl alcohol) hydrogel. RSC Adv..

[70] Chen Y., Song J., Wang S., Liu W. (2021). PVA-Based Hydrogels: Promising Candidates for Articular Cartilage Repair. Macromol. Biosci..

[71] Gaharwar A.K., Schexnailder P.J., Schmidt G., Sitharaman B. (2011). Nanocomposite Polymer Biomaterials for Tissue Repair of Bone and Cartilage. Nanobiomaterials Handbook.

[72] Little C.J., Bawolin N.K., Chen X. (2011). Mechanical Properties of Natural Cartilage and Tissue-Engineered Constructs. Tissue Eng. Part B Rev..

[73] Tamaddon M., Wang L., Liu Z., Liu C. (2018). Osteochondral tissue repair in osteoarthritic joints: Clinical challenges and opportunities in tissue engineering. Bio-Design Manuf..

[74] Athanasiou K.A., Darling E.M., DuRaine G.D., Hu J.C., Reddi A.H. (2013). Articular Cartilage.

[75] Doulabi A.H., Mequanint K., Mohammadi H. (2014). Blends and Nanocomposite Biomaterials for Articular Cartilage Tissue Engineering. Materials.

[76] Stojkov G., Niyazov Z., Picchioni F., Bose R.K. (2021). Relationship between Structure and Rheology of Hydrogels for Various Applications. Gels.

[77] Dehghan-Niri M., Vasheghani-Farahani E., Eslaminejad M.B., Tavakol M., Bagheri F. (2020). Physicomechanical, rheological and in vitro cytocompatibility properties of the electron beam irradiated blend hydrogels of tyramine conjugated gum tragacanth and poly (vinyl alcohol). Mater. Sci. Eng. C.

[78] Moore A., Burris D. (2017). Tribological rehydration of cartilage and its potential role in preserving joint health. Osteoarthr. Cartil..

[79] Yang X., Zhu Z., Liu Q., Chen X., Ma M. (2008). Effects of PVA, agar contents, and irradiation doses on properties of PVA/ws-chitosan/glycerol hydrogels made by γ-irradiation followed by freeze-thawing. Radiat. Phys. Chem..

[80] Zainuddin, Cooper-White J.J., Hill D.J.T. (2002). Viscoelasticity of radiation-formed PVA/PVP hydrogel. J. Biomater. Sci. Polym. Ed..

[81] Mostakhdemin M., Nand A., Ramezani M. (2021). Articular and Artificial Cartilage, Characteristics, Properties and Testing Approaches—A Review. Polymers.

[82] Boettcher K., Grumbein S., Winkler U., Nachtsheim J., Lieleg O. (2014). Adapting a commercial shear rheometer for applications in cartilage research. Rev. Sci. Instrum..

[83] Link J.M., Salinas E.Y., Hu J.C., Athanasiou K.A. (2020). The tribology of cartilage: Mechanisms, experimental techniques, and relevance to translational tissue engineering. Clin. Biomech..

[84] Feng Y., Dai S.-C., Lim K., Ramaswamy Y., Jabbarzadeh A. (2022). Tribological and Rheological Properties of Poly(vinyl alcohol)-Gellan Gum Composite Hydrogels. Polymers.

[85] Gong J.P. (2006). Friction and lubrication of hydrogels—Its richness and complexity. Soft Matter.

[86] Gong J., Iwasaki Y., Osada Y., Kurihara K., Hamai Y. (1999). Friction of Gels. 3. Friction on Solid Surfaces. J. Phys. Chem. B.

[87] Chen K., Zhang D., Cui X., Wang Q. (2015). Research on swing friction lubrication mechanisms and the fluid load support characteristics of PVA–HA composite hydrogel. Tribol. Int..

[88] McCutchen C. (1962). The frictional properties of animal joints. Wear.

[89] Brand R.A. (2005). Joint contact stress: A reasonable surrogate for biological processes?. Iowa Orthop. J..

[90] Noguchi T., Yamamuro T., Oka M., Kumar P., Kotoura Y., Hyonyt S., Ikadat Y. (1991). Poly(vinyl alcohol) hydrogel as an artificial articular cartilage: Evaluation of biocompatibility. J. Appl. Biomater..

[91] Ali M., Al-Hajjar M., Partridge S., Williams S., Fisher J., Jennings L.M. (2016). Influence of hip joint simulator design and mechanics on the wear and creep of metal-on-polyethylene bearings. Proc. Inst. Mech. Eng. Part H J. Eng. Med..

[92] Inês M., De Sá M. (2019). Tribomechanical Behaviour of Chemically Crosslinked PVA Hydrogels Bioengineering and Nanosystems. Master’s Thesis.

[93] Henke P., Ruehrmund L., Bader R., Kebbach M. (2024). Exploration of the Advanced VIVO^TM^ Joint Simulator: An In-Depth Analysis of Opportunities and Limitations Demonstrated by the Artificial Knee Joint. Bioengineering.

[94] Li J., Wang C., Han X., Liu S., Gao X., Guo C., Wu X. (2022). Aramid Nanofibers-Reinforced Rhein Fibrous Hydrogels as Antibacterial and Anti-Inflammatory Burn Wound Dressings. ACS Appl. Mater. Interfaces.

[95] (2009). Biological Evaluation of Medical Devices—Part 5: Tests for In Vitro Cytotoxicity.

[96] Jimenez-Cruz D., Dubey M., Board T., Williams S. (2022). An in vitro methodology for experimental simulation on the natural hip joint. PLoS ONE.

[97] (2014). Implants for Surgery—Wear of Total Hip-Joint Prostheses—Part 1: Loading and Displacement Parameters for Wear-Testing Machines and Corresponding Environmental Conditions for Test.

[98] (2012). Biological Evaluation of Medical Devices—Part 12: Sample Preparation and Reference Materials.

