# Supplementary material for: Tribomechanical Properties of PVA/Nomex® Composite Hydrogels for Articular Cartilage Repair
Source: Gels. 2024 Aug 3;10(8):514. doi: 10.3390/gels10080514 (PMC11354222; doi:10.3390/gels10080514)
Supplement: Supplementary file 1 [file gels-10-00514-s001.zip › gels-3101727-supplementary.pdf]

SUPPLEMENTARY INFORMATION

Tribomechanical properties of PVA/Nomex® composite hydrogels for articular cartilage repair

Francisco Santos [a], Carolina Marto-Costa [b,c], Ana Catarina Branco [a,b,c], Andreia Sofia Oliveira [a,b,d], Rui Galhano dos Santos [e], Madalena Salema-Oom [b], Roberto Leonardo Diaz [f], Sophie Williams [f], Rogério Colaço [d], Célio Figueiredo-Pina [b,c,g *], and Ana Paula Serro [a,b*]

[a] Centro de Química Estrutural (CQE) - Institute of Molecular Sciences and Department of Chemical Engineering, Instituto Superior Técnico, University of Lisbon, Av. Rovisco Pais 1, 1049-001 Lisbon, Portugal;

[b] Egas Moniz Center for Interdisciplinary Research (CiiEM); Egas Moniz School of Health & Science, 2829-511 Monte da Caparica, Almada, Portugal;

[c] CDP2T, Escola Superior de Tecnologia de Setúbal, Instituto Politécnico de Setúbal, 2910-761 Setúbal, Portugal;

[d] Instituto de Engenharia Mecânica (IDMEC) and Department of Mechanical Engineering, Instituto Superior Técnico, Universidade de Lisboa,

Av. Rovisco Pais 1, 1049-001 Lisbon, Portugal;

[e] CERENA - Centre for Natural Resources and the Environment, Instituto Superior Técnico, Av. Rovisco Pais, 1049-001 Lisboa, Portugal;

[f] Institute of Medical And Biological Engineering, School of Mechanical Engineering, University of Leeds, Woodhouse, Leeds LS2 9JT, United Kingdom;

[g] CeFEMA, Instituto Superior Técnico, Universidade de Lisboa, Av. Rovisco Pais 1, 1049-001 Lisbon, Portugal

* Correspondence: anapaula.serro@tecnico.ulisboa.pt; celio.pina@estsetubal.ips.pt

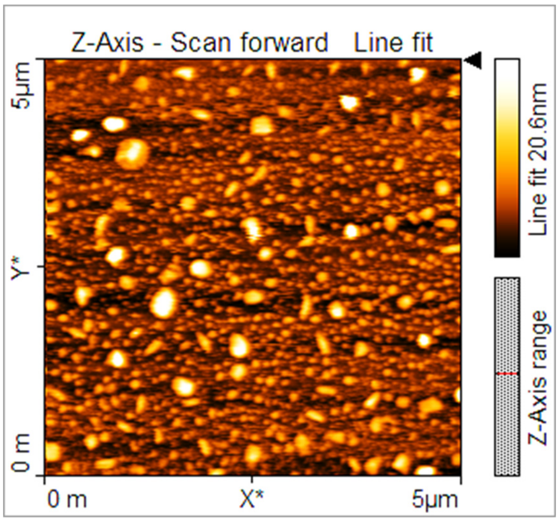

Figure S1. AFM image of the PMIA nanoparticles.

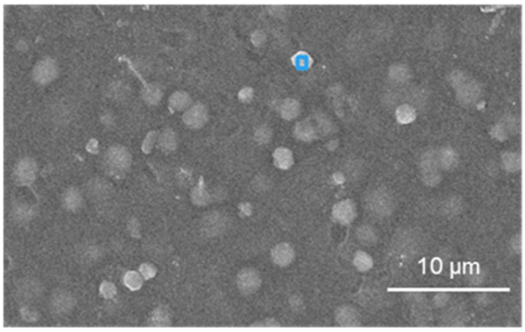

Element	Atomic Concentration [%]	Weight Concentration [%]
Carbon	51.2	45.4
Nitrogen	20.2	20.9
Oxygen	28.6	33.8

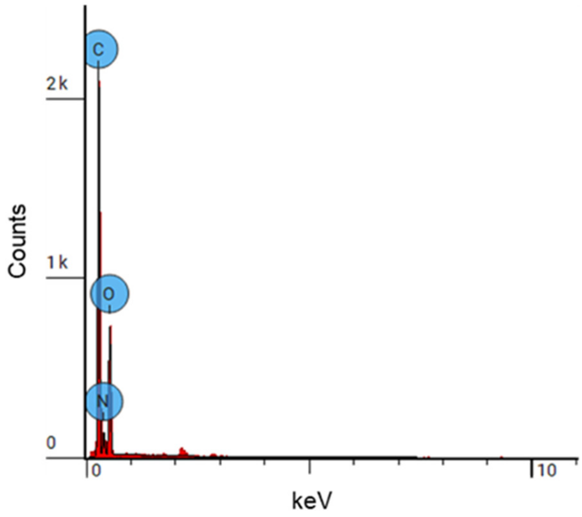

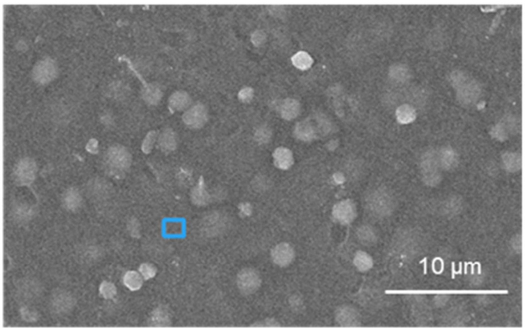

Element	Atomic Concentration [%]	Weight Concentration [%]
Carbon	67.1	61.2
Nitrogen	7.3	7.8
Oxygen	25.5	31.0

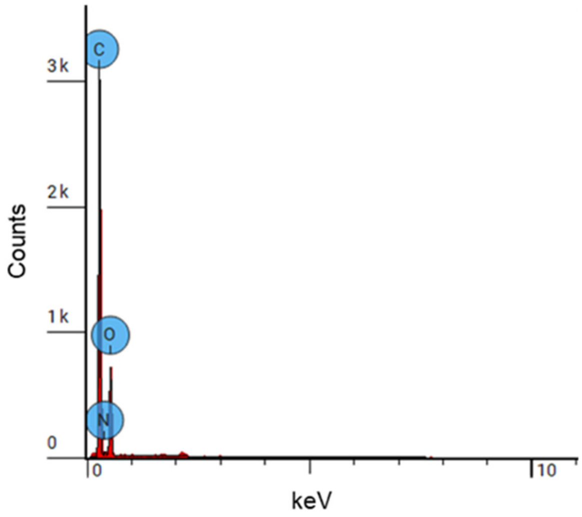

Figure S2. EDS analysis of the spherical structures within the reinforced hydrogels (left) compared to the surrounding material (right). The data presented are from the analysis of the sample PVA10/PMIA1.5_G.

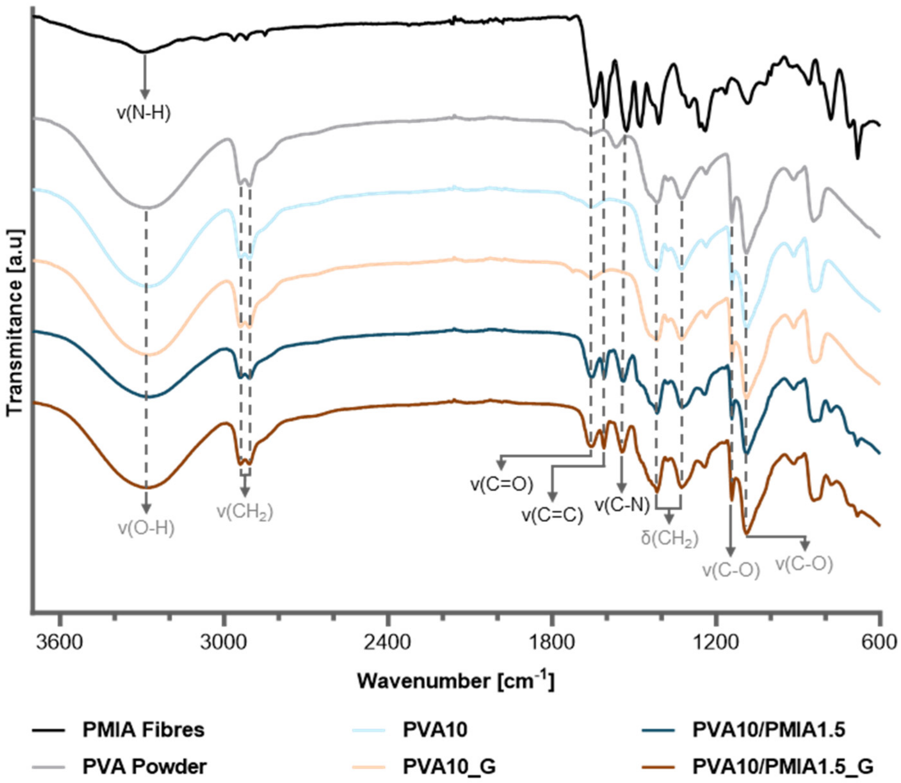

Figure S3. ATR-FTIR spectra of commercial PMIA fibers, commercial PVA powder, and hydrogels PVA10 and PVA10/PMIA1.5, before and after sterilization.

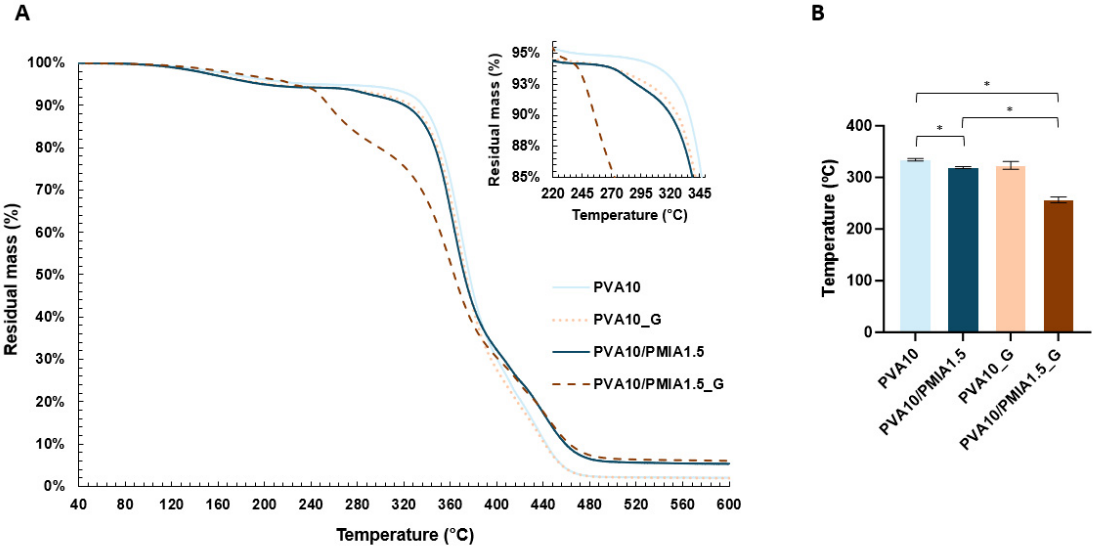

Figure S4. TGA curves of irradiated and non-irradiated PVA10 and PVA10/PMIA1.5.

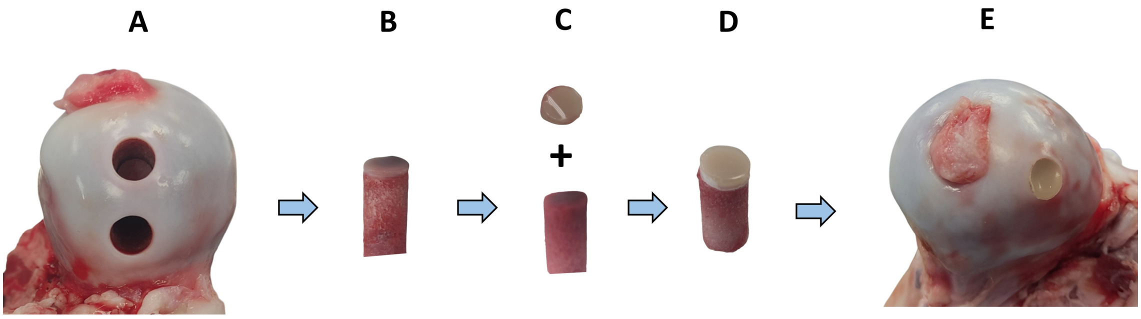

Figure S5. Schematic representation of the hydrogel-modified bone plug insertion procedure.
